# A novel *Filamentous Flower* mutant suppresses *brevipedicellus* developmental defects and modulates glucosinolate and auxin levels

**DOI:** 10.1371/journal.pone.0177045

**Published:** 2017-05-11

**Authors:** Scott J. Douglas, Baohua Li, Daniel J. Kliebenstein, Eiji Nambara, C. Daniel Riggs

**Affiliations:** 1 Department of Biological Sciences, University of Toronto-Scarborough, Scarborough, Ontario, Canada; 2 Department of Plant Sciences, University of California Davis, Davis, California, United States of America; 3 DynaMo Center of Excellence, Copenhagen Plant Science Centre, University of Copenhagen, Denmark; 4 Department of Cell and Systems Biology, University of Toronto, Toronto, Ontario, Canada; 5 Centre for the Analysis of Gene Evolution and Function, University of Toronto, Toronto, Ontario, Canada; Instituto de Biologia Molecular y Celular de Plantas, SPAIN

## Abstract

*BREVIPEDICELLUS* (*BP*) encodes a class-I *KNOTTED1*-like homeobox (*KNOX*) transcription factor that plays a critical role in conditioning a replication competent state in the apical meristem, and it also governs growth and cellular differentiation in internodes and pedicels. To search for factors that modify BP signaling, we conducted a suppressor screen on *bp er (erecta*) plants and identified a mutant that ameliorates many of the pleiotropic defects of the parent line. Map based cloning and complementation studies revealed that the defect lies in the *FILAMENTOUS FLOWER (FIL)* gene, a member of the YABBY family of transcriptional regulators that contribute to meristem organization and function, phyllotaxy, leaf and floral organ growth and polarity, and are also known to repress *KNOX* gene expression. Genetic and cytological analyses of the *fil-10* suppressor line indicate that the role of FIL in promoting growth is independent of its previously characterized influences on meristem identity and lateral organ polarity, and likely occurs non-cell-autonomously from superior floral organs. Transcription profiling of inflorescences revealed that FIL downregulates numerous transcription factors which in turn may subordinately regulate inflorescence architecture. In addition, FIL, directly or indirectly, activates over a dozen genes involved in glucosinolate production in part by activating *MYB28*, a known activator of many aliphatic glucosinolate biosynthesis genes. In the *bp er fil-10* suppressor mutant background, enhanced expression of *CYP71A13*, *AMIDASE1 (AMI)* and *NITRILASE* genes suggest that auxin levels can be modulated by shunting glucosinolate metabolites into the IAA biosynthetic pathway, and increased IAA levels in the *bp er fil-10* suppressor accompany enhanced internode and pedicel elongation. We propose that FIL acts to oppose *KNOX1* gene function through a complex regulatory network that involves changes in secondary metabolites and auxin.

## Introduction

Growth and development of terrestrial plants is guided by events occurring at meristems, zones where pluripotent stem cells perpetuate themselves and generate raw material for organ production. For aerial development, the shoot apical meristem (SAM) elaborates leaf, stem and flower anlagen at specific regions depending on complex temporal and spatial interactions between proteins, microRNAs and hormones [1,2]. The SAM shares common mechanisms of regulation with floral meristems, which form during the reproductive phase to generate sepals, petals, stamens and carpels, with an important difference being that floral meristems are determinate.

Genes affecting SAM and floral meristem patterning, maintenance, and function have been identified by both forward and reverse genetic screens. One family of genes that plays a prominent role in promoting meristem function throughout the plant life cycle is the class I *KNOTTED*-like homeobox (*KNOX1*) genes, which were named for the founding member, *KNOTTED1* (*KN1*) from maize (reviewed in [3]). Leaf blades of the *kn1* dominant mutant display knots of undifferentiated cells around lateral veins due to ectopic expression of the *KN1* gene product [4,5]. In numerous monocot and dicot species, the expression of a variety of *KNOX1* proteins in leaves conditions the production of ectopic meristems, implicating the factors as critical regulators of meristem function in a diverse array of plants [6–8].

In addition to their role in meristems, *KNOX1* genes promote growth in aerial organs such as leaves, flowers and stems. For example, compound leaves of tomato are observed to branch and form supercompound leaves if either the *LeT6 KNOX* gene or the maize *KN1* gene is ectopically expressed [9]. In tobacco, maize and *Arabidopsis*, ectopic expression of *KNOX1* genes also results in alterations in leaf architecture [6, 8–13]. In rice and *Arabidopsis*, *KNOX1* genes are known to promote both longitudinal and radial growth of stems [14–16].

A large number of factors interact with *KNOX1* genes to influence meristem and organ growth and morphology (reviewed in [17]). KNOX1 proteins promote cytokinin biosynthesis to sponsor meristematic activity and cell division [18–20] and conversely, repress gibberellin function in meristems to support meristem maintenance [12, 21–22]. In many cases, *KNOX1* genes are expressed in meristems but are downregulated as lateral organs are initiated, but they can be reactivated in compound leaf species [23]. Families of genes that encode the adaxializing factors *ASYMMETRIC LEAVES1 (AS1)* and *ASYMMETRIC LEAVES2 (AS2)* in *Arabidopsis* [24–26], *PHANTASTICA* in *Antirhinnum* and other species [27–28], and *ROUGHSHEATH2* in maize [29–30] repress *KNOX* genes as well as genes encoding some of the abaxializing factors of the *YABBY* and *KANADI* families in leaf primordia (reviewed in [31]). In addition, some *YABBY* proteins play roles in negatively regulating *KNOX* genes in lateral organs [32]. Collectively, these antagonistic interactions assist in establishing distinct domains of gene expression that promote proper lateral organ polarity. In contrast to these well-established examples of hierarchical controls that pattern leaves, little is known of the factors that act coordinately with *KNOX1* genes in stems to control morphogenesis.

We have previously characterized the expression and function of the *Arabidopsis KNOX1* gene *BREVIPEDICELLUS* (*BP*), which is required to promote elongation and radial expansion of inflorescence stems and pedicels, short stems that orient flowers and siliques at an upright angle along inflorescences [15]. BP acts in a partially redundant manner with the ERECTA (ER) receptor protein kinase, as double mutant *bp er* pedicels develop downward bends that are due to growth suppression on the abaxial side. Pedicel abnormalities of the *bp er* mutant are spatially linked to the patterning of underlying vascular bundles that are continuous with associated floral organs, and nodal identity is translated downwards into subtending internodes [33]. This stimulated the hypothesis that *BP* and *ER* promote growth along pedicels and internodes at least in part by counteracting growth-repressive signals that originate from superior organs and are borne by the vasculature. To explore this further, we conducted a suppressor screen of *bp er*, and identified a point mutation in the *FILAMENTOUS FLOWER* (*FIL*) gene that suppresses many of the *bp er* pleiotropic phenotypes. *FIL* is a member of the *YABBY* family of transcriptional regulators, which play roles in leaf and floral organ polarity, organ growth, phyllotaxy and shoot apical meristem organization and function [34–43]. Our analyses indicate that the effect of the *fil-10* suppressor mutation on pedicel development is also due to mobile signaling from the flower, and is not linked to the role of *FIL* in promoting abaxial organ fate. Subsequent microarray analyses revealed that numerous genes encoding glucosinolate (GSL) biosynthetic enzymes are repressed in the *fil-10* suppressor, and the levels of many glucosinolate metabolites are significantly reduced. These changes in GSL levels are correlated with elevated auxin levels that likely influence inflorescence architecture.

## Materials and methods

### Biological materials

Unless otherwise stated the parent background was *bp-2 er*, for which extensive phenotypic and molecular analyses have been conducted [15, 33]. The *fil-2*, *fil-3*, *fil-4* and *fil-5* alleles were obtained from Dr. Gary Drews. The mutant alleles for *ap1-1* (CS28), *as2-101* (CS16274), and *lug-1* (CS3081) were obtained from the Arabidopsis Biological Resource Center. Wildtype Landsberg Lan (La-1) was obtained from Dr. Detlef Weigel. Seeds of *kan1-2*, *kan2-1*, *las-11* and *yab3-2* mutants were obtained from Dr. John Bowman. Double and higher order mutants were constructed by crosses and validated by either visual phenotypes conferred by the mutant, and/or molecular genotyping (CAPS analysis where possible; direct sequencing for others as is described in S1 Table).

### EMS mutagenesis and plant growth conditions

Approximately 10,000 *bp-2 er* seeds were placed in 50ml of 0.2% EMS (Aldrich) for 16h. Seeds were washed extensively with water and planted in 20cm plastic pots in Premier Promix PGX at a density of approximately 200 seeds per pot. M1 plants were grown under natural lighting conditions in a greenhouse. M2 seeds were regrown in Conviron growth chambers at 22°C under fluorescent lighting (125**μ**E/m^2^) with a 16hr day:8hr night photoperiod.

### Microscopy and morphometric analyses

Light, SEM and fluorescence microscopy were carried out as previously described [33]. Morphometric measurements were conducted with mature plants and as previously described [33]. For pedicel measurements, samples were taken from the lower nodes of the plant to ensure the acropetal gradient of development was not a complicating issue. *In situ* hybridizations were performed as described in Lincoln *et al*. [44]. For confocal microscopy of FIL::GFP plants, buds of 0.3 to 0.5mm were dissected and embedded in 4% agarose. The blocks were affixed to a sectioning plate with superglue and 80-120um sections cut using a Leica VT1000S vibratome. Sections were mounted in cold water and imaged using a Zeiss 510 Meta laser scanning confocal microscope with an excitation wavelength of 488nm and a pinhole adjustment of 1.74–1.81 Airy units. An emission bandpass filter of 510-530nm was used to collect GFP fluorescence. Images were edited using LSM image Browser software, version 3 (Zeiss).

### Mapping of the *fil-10* suppressor mutant

*bp er fil-10*, backcrossed twice to *bp er*, was crossed with Columbia harboring a mutation in the *ER* gene (C*er*) to establish a mapping population. DNA from F_2_ plants displaying a *bp er fil-10* or *er fil-10* phenotype was used for simple sequence length polymorphisms (SSLPs) or dCAPS analyses [45]. For additional experiments involving microarray analysis and glucosinolate/auxin profiling, two additional backcrosses to the *bp er* parent line were performed.

### Identification of the lesion in *fil-10*

The *FIL* gene was amplified by PCR from *bp er fil-10* and *bp er* genomic DNA using FIL FOR: 5’ AAAAGATGTCTATGTCGTCTATGTCCTCC 3’ and FIL BACK: 5’ GAATCGGTTATATGCGGATGGGACTC 3’ primers. PCR products were gel purified (Qiagen) and both strands were sequenced using the *FIL* F/B primers (S1 Table). To validate sequencing results, the procedure was repeated on a second series of plants, and gave identical results.

### Transgenic construction and analysis

To examine FIL protein localization, a GFP tagged version was generated by amplifying the FIL promoter (2.7kb) and coding region with the primers: FIL/GFP FOR 5’TCGGAGCTCGATTCTTCATATGTTAAGTTATGCTGA 3’ and FIL/GFP BACK 5’TAACCGGTGCAGGAGCGTAGAACCCTTCTTTCATCACC 3’ using Phusion (New England Biolabs) polymerase. These primers engineer 5’ Sac I and 3’ Age I sites to facilitate cloning into pEGAD. Sequencing confirmed an in-frame fusion of *FIL* with *GFP*, where the last eight amino acids of FIL are missing. The construct was mobilized into *Agrobacterium* strain GV3101, and used to transform *bp fil-10 er* plants via the floral dip procedure [46]. Transgenics were selected on 0.5X MS media containing 10**μ**g/ml BASTA (Crescent Chemicals).

### Microarray analyses and QRT-PCR

Inflorescences from five-week old plants were used as a source of total RNA for both microarray analyses and QRT-PCR. Older flowers were culled from the periphery of the inflorescence such that no buds of later than stage 13 (bud opening defined by Smyth et al. [47] were used. For microarray analysis, total RNA was prepared from inflorescences of *bp er* and *bp er fil-10* plants in triplicate, using the Qiagen RNeasy system. RNA was reverse transcribed into cDNA pools using oligo dT, and the cDNA was amplified by in vitro transcription with biotinylated CTP to generate probes. Affymetrix ATH1 arrays were employed, and hybridization and washing conditions were carried out as described by the manufacturer. Detection/quantitation was facilitated by using an Affymetrix GeneChip scanner 3000. Raw data was subjected to GCOS/MAS normalization and a linear scaling factor was applied to set the TGT value to 500. The list was culled by discarding genes for which values were low and hence were called ‘absent’. Lists of UP/DOWN regulated genes were then obtained by sorting the Excel spreadsheet. Individual values from the triplicate samples were then examined and genes were removed from the list if the average value was skewed by an anomalous signal. Cutoff values were arbitrarily set at 2.5 fold and 1.9 fold to generate short and extended lists of genes influenced by *FIL*. Raw data and additional information can be accessed through the GEO accession number GSE86643. Analyses are presented in S2 and S3 Tables.

For QRT-PCR, total RNA was prepared as described above, and on-column DNAse digestion was undertaken, using RNAse free DNAse I (Invitrogen). cDNA pools were generated by reverse transcription of 1ug of total RNA, employing oligo dT as a primer and Superscript III reverse transcriptase (Invitrogen). An MJ Research instrument was used to amplify cDNAs to validate the microarray results and to test other putative target genes, using Sensifast SYBR mix (Bioline). Primers were designed by employing the open source Primer3 software. Primer efficiency tests were performed on dilutions of cDNA, and melting curves and gel analysis used to confirm primer specificity. Several potential reference genes were tested with both *bp er* and *bp er fil* cDNAs to determine the most reliable set. PP2a (At4g15415) and ACT7 (At5g09810) exhibited minimal variation and their primer efficiencies (E) and ΔCT values were averaged for normalization of target gene data. The relative expression ratio was calculated as described by Pfaffl [48], and pairwise type three Student’s t-tests conducted by transforming ΔCT values to linear terms by the equation (1+E)^− ΔCT^ as described by Livak and Schmittgen [49]. Two independent biological experiments that employed three to four technical replicates were carried out for each primer set. The independent experiment is summarized in S1 Fig. A list of primers is provided in S1 Table.

### Glucosinolate and auxin profiling

Inflorescences were dissected from five week old plants, their fresh weights recorded, and then placed in either 100% methanol (for glucosinolate profiling), or a solution of 80% methanol, 1% acetic acid (for IAA determination). Glucosinolate metabolites were identified and quantitated by HPLC as described by Kliebenstein *et al*. [50], and IAA levels were determined as described by Stokes *et al*. [51]. For IAA measurements, two independent experiments were carried out and revealed similar trends, and three experiments were conducted to profile glucosinolate metabolites, which also showed similar trends.

### DR5::GUS analysis in *bp er* and *bp er fil-10* genetic backgrounds

The DR5::GUS cassette was resected from a pBIN19 derivative with Sal I and EcoRI, and recloned into the Xho I/ EcoRI sites of pEGAD-link in order to use BASTA as a selectable marker. Following validation of primary transformants, T2 seeds were surface sterilized and germinated on media containing 0.5XMS salts, 5mM MES pH 5.7, 1% sucrose, and 10**μ**g/ml BASTA. Ten day old seedlings were fixed in 90% acetone for 30 minutes on ice, followed by one wash each in cold water and x-gluc buffer (50mM phosphate buffer, pH7.2, 0.2% Triton X-100, 2mM potassium ferrocyanide, 2mM potassium ferricyanide). X-gluc buffer containing 1mM x-gluc (BioShop Canada) was added and seedlings were incubated in the dark at room temperature for 8 hours, then fixed/decolorized with an ethanol series. The alcohol was exchanged for 8:2:1 chloral hydrate:glycerol:water and following overnight incubation at 4°C, slides of individual seedlings were prepared, coverslipped, and photographed using a Nikon SMZ1500 stereomicroscope with a digital imaging system (Nikon Digital Sight D5 Fi1). To investigate DR5 copy number in the transgenic lines, multiplex PCR was employed using the primers EGADjunctionFOR/GUS genotype back to screen for DR5::GUS insertions, and AMIgenotypeFOR/AMIgenotypeBACK as a single copy gene control. Primer sequences and PCR conditions are given in S1 Table.

## Results

### Identification of *fil-10* as a suppressor of *bp er* phenotypes

**Fig 1:**
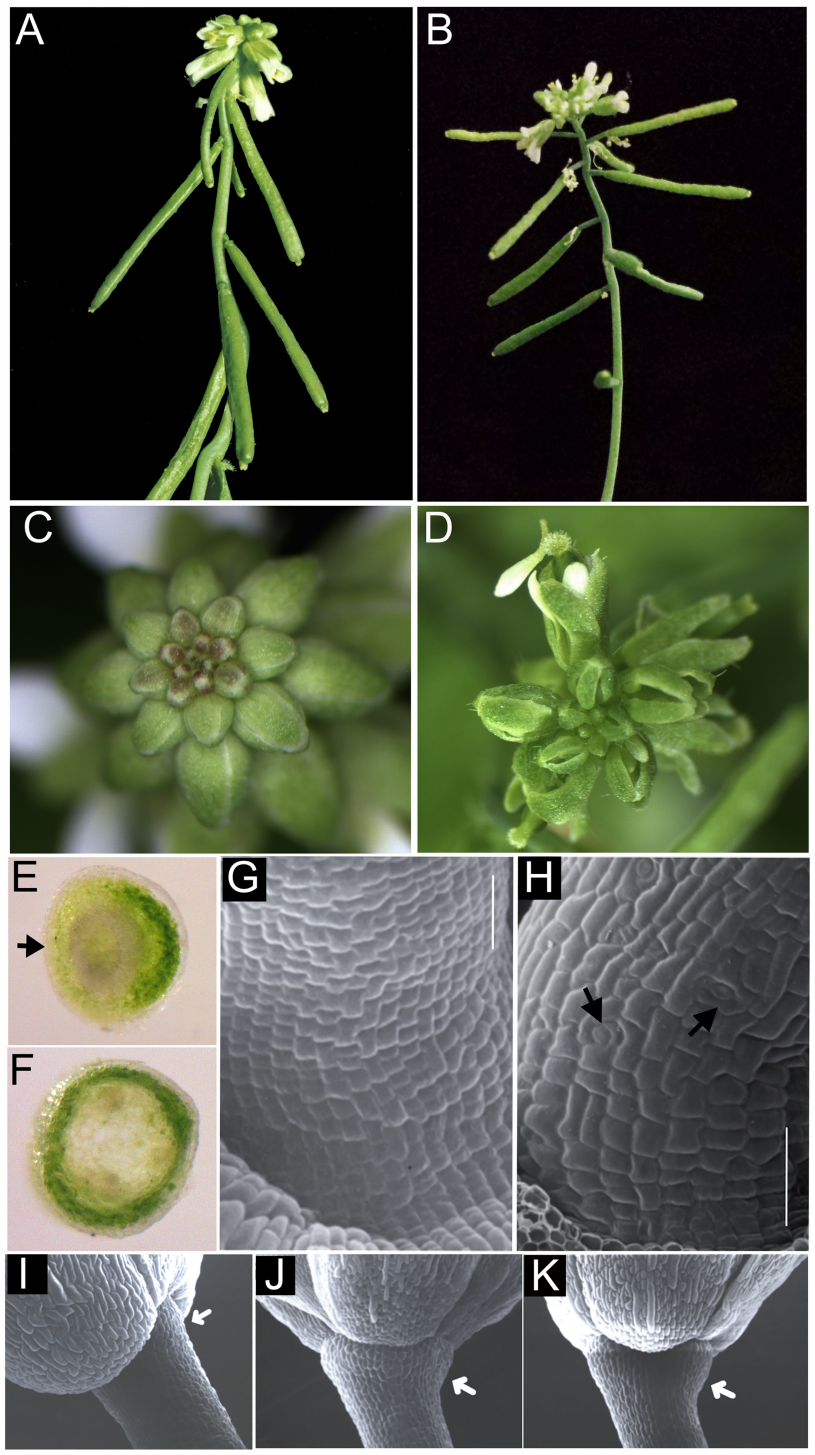
Suppression of *bp er* pedicel phenotypes by the *fil-10* mutation. A *bp er* plant showing short pedicels that bend downwards. (B) *bp er fil-10* plant exhibiting enhanced internode growth and elongated pedicels perpendicular to the stem axis. The acute pedicel angle defect is partially ameliorated. (C) *bp er* inflorescence cluster with closed floral buds. (D) Young *bp er fil-10* flowers with visible inner whorl organs due to aberrant sepal development. (E) Hand section of a *bp er* pedicel. Note the lack of chlorenchyma development on the abaxial side (arrow). (F) Hand section of a *bp er fil-10* pedicel, revealing a continuous ring of chlorenchyma tissue. (G) The *bp er* pedicels display files of short cells on their abaxial sides and differentiation of guard cells is repressed. (H) In *bp er fil-10*, the pedicel stripe is confined to a narrow band of stomata free tissue on lateral sides, but abaxial cells are larger and assume the irregular shapes found in wild type. Differentiation of stomata is also observed (arrows) (I-K) Receptacles of *bp er fil-10* (I), *fil-10 er* (J) and L*er* (K). Note expansion in *fil-10 er* and L*er* but lack of enlargement in *bp er fil-10* (arrow). Bars in panels G and H are 50 **μ**M.

Wild-type *Arabidopsis* pedicels elongate as straight stems to support flowers and siliques at an upright angle along inflorescence axes. In *bp er* mutants, pedicel elongation is compromised and pedicels acquire bends that orient flowers at a downward angle (Fig 1A). To identify other genes controlling pedicel development, *bp er* seeds were mutagenized with EMS, and an M_2_ plant with elongated, perpendicular pedicels was identified (Fig 1B) and backcrossed to *bp er*. F_2_ plants segregated the suppressed phenotype in a 3:1 ratio, demonstrating that the novel phenotype is due to a recessive mutation at a single locus. Examination of unopened flowers with a dissecting microscope revealed narrow sepals that failed to fully conceal developing inner reproductive organs (Fig 1C and 1D). Further genetic and molecular characterization (see below) demonstrated allelism between the suppressor mutant and the *FILAMENTOUS FLOWER (FIL)* gene, and hereafter we refer to the mutant as *fil-10*.

Light microscopy of hand sections of pedicels showed that, in contrast to the disruptions of chlorenchyma tissue associated with the abaxial side of *bp er* pedicels (Fig 1E; [33]), *bp er fil-10* pedicels displayed a continuous ring of chlorenchyma (Fig 1F). Similarly, while the epidermis of *bp er* pedicels exhibits files of short cells that lack stomata on abaxial and lateral sides (Fig 1G), this feature is strongly suppressed in *bp er fil-10*, which exhibits a relatively indistinct stripe of undifferentiated cells along the lateral sides, and a more wild-type array of irregularly shaped cells on other sides. In contrast to the *bp er* line, the pedicels of the suppressor line also differentiate guard cells on all sides (Fig 1H). Our previous work demonstrated that BP plays a role in receptacle enlargement as gauged by a constriction of tissue at the distal end of the pedicel in *bp* mutants [33]. However, unlike the suppression of other defects, the *bp er fil-10* receptacles did not enlarge as they did the *fil-10 er* or L*er* plants (Fig 1I–1K). Receptacle growth is enhanced by overexpression of BP [33] and our results indicate that the mechanism controlling pedicel morphogenesis is genetically separable from that regulating receptacle growth. While FIL contributes to growth and patterning of stems, pedicels and floral organs, it apparently does not play a role in receptacle enlargement.

**Fig 2:**
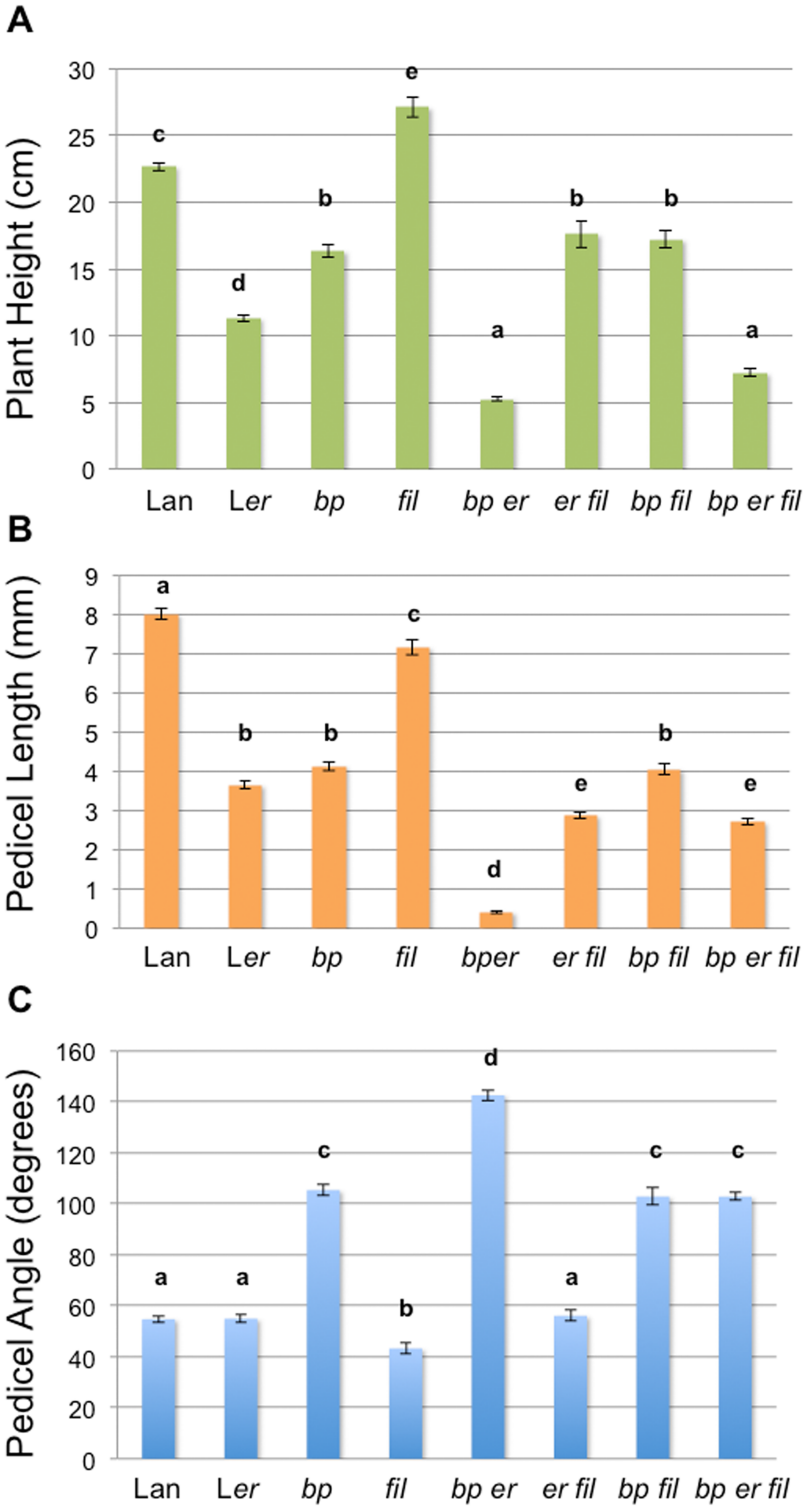
Morphometric analyses and differential effects of mutations in *bp*, *er*, and/or *fil*. Crosses were used to generate all combinations of single, double and triple mutants in a Landsberg (Lan) background. (A) Plant height was measured from the rosette to the inflorescence tip in six week old plants. (B-C) Mature, senescing plants were used to measure pedicel length (B) and angle (C). The error bars represent standard error of the mean. Data were compared by one way ANOVA using Tukey’s Honest Significant Differences method. Letters above the bars indicate significance categories where p< 0.01. For all measurements, n = 15–150. Similar trends were observed in two independent experiments.

Developmental analyses of *bp er fil-10* plants showed that *bp er* pedicel phenotypes are increasingly suppressed as development progresses (2.5mm ± 0.1mm pedicel length (pl); 108° ± 2° pedicel angle (pa) for flowers 1–5 and 2.9mm ± 0.1mm pl; 98° ± 2° pa for flowers 6–10). To examine interactions between *fil-10*, *bp* and *er*, height, pedicel length and pedicel projection angle comparisons were made between all possible genotypes. Relative to the baseline genotype Landsberg, mutations in both *BP* and *ER* result in compromised internode elongation, while *fil-10* enhances growth (Fig 2A). These relationships are supported by the double mutant phenotypes in which either *bp* or *er* in combination with *fil-10* conditions less robust growth than *fil-10* alone. The effect on plant height is less pronounced when *bp er* is compared with the triple *bp er fil* mutant. Pedicel growth is also affected by the three genes in a manner similar to internode elongation (Fig 2B). The *bp* mutation significantly alters the pedicel angle and the angle becomes more pronounced by combining *bp* with *er*. The *fil-10* mutation suppresses this effect, giving rise to perpendicular pedicels in the triple mutant (Fig 2C). In summary, the *fil-10* suppressor partially ameliorates the *bp er* defects in internode and pedicel elongation, and conditions differential growth and development of pedicels to alter plant architecture.

### Characterization of *fil-10* floral phenotypes

The *fil-10* suppressor line exhibits reduced fecundity, producing short siliques with fewer viable seeds that may be due to reduced levels or viability of pollen. We assessed female viability by crossing L*er* pollen into *fil-10 er* gynoecia. Siliques elongated and set seed, indicating that either an anther or male gametophyte defect underlies reduced *fil-10* fertility. To distinguish between sporophytic and gametophytic possibilities, *fil-10/+ er* pollen was crossed into L*er* gynoecia. The F_1_ plants appeared normal and were fully fertile. Genotyping revealed that 27/62 = 43.5% of plants were heterozygous for *fil-10*, consistent with the 50% value expected if *fil-10* and wild-type pollen grains are equally viable. Thus, reduced seed set is due to a sporophytic defect probably related to low pollen yield.

**Fig 3:**
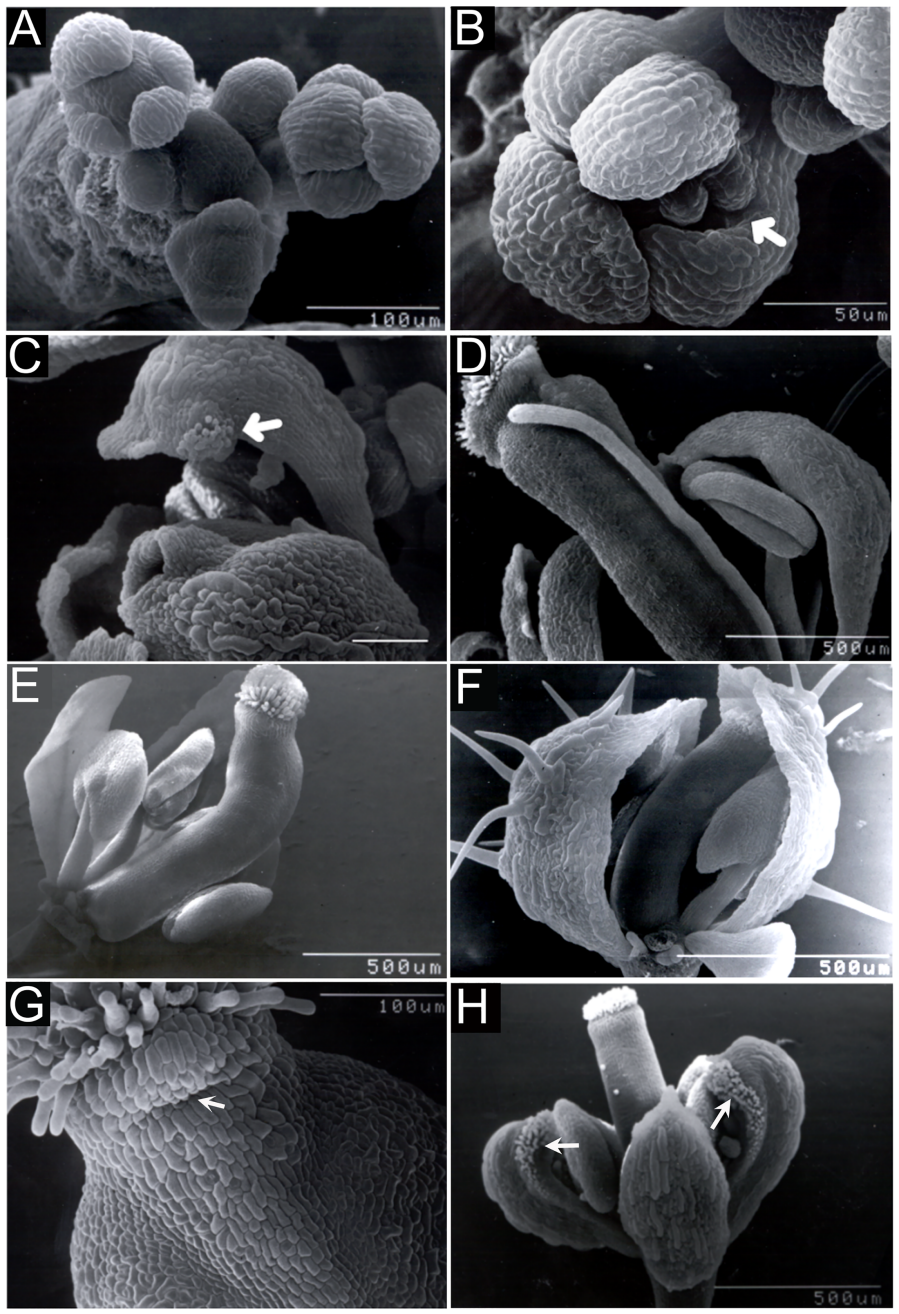
*fil-10* conditions floral organ abnormalities. (A-G) *fil-10 er* flowers. (A) Early inflorescences showing symmetrically located sepal primordia. (B) An early bud with a gap (arrow) between two sepals. (C) A flower formed late in development with stigmatic tissue (arrow) on the tip of a sepal. (D) A flower with a third whorl filament lacking an anther. (E) A gynoecium with a bend. (F) A gynoecium in the midst of bending due to sustained contact with the inner face of a lateral sepal. (G) Medial region of a gynoecium showing a bulge of style tissue (arrow) under the stigma. (H) *fil-10 ap1-1 er* flower showing transformation of medial sepals (arrows) into carpelloid organs.

**Fig 4:**
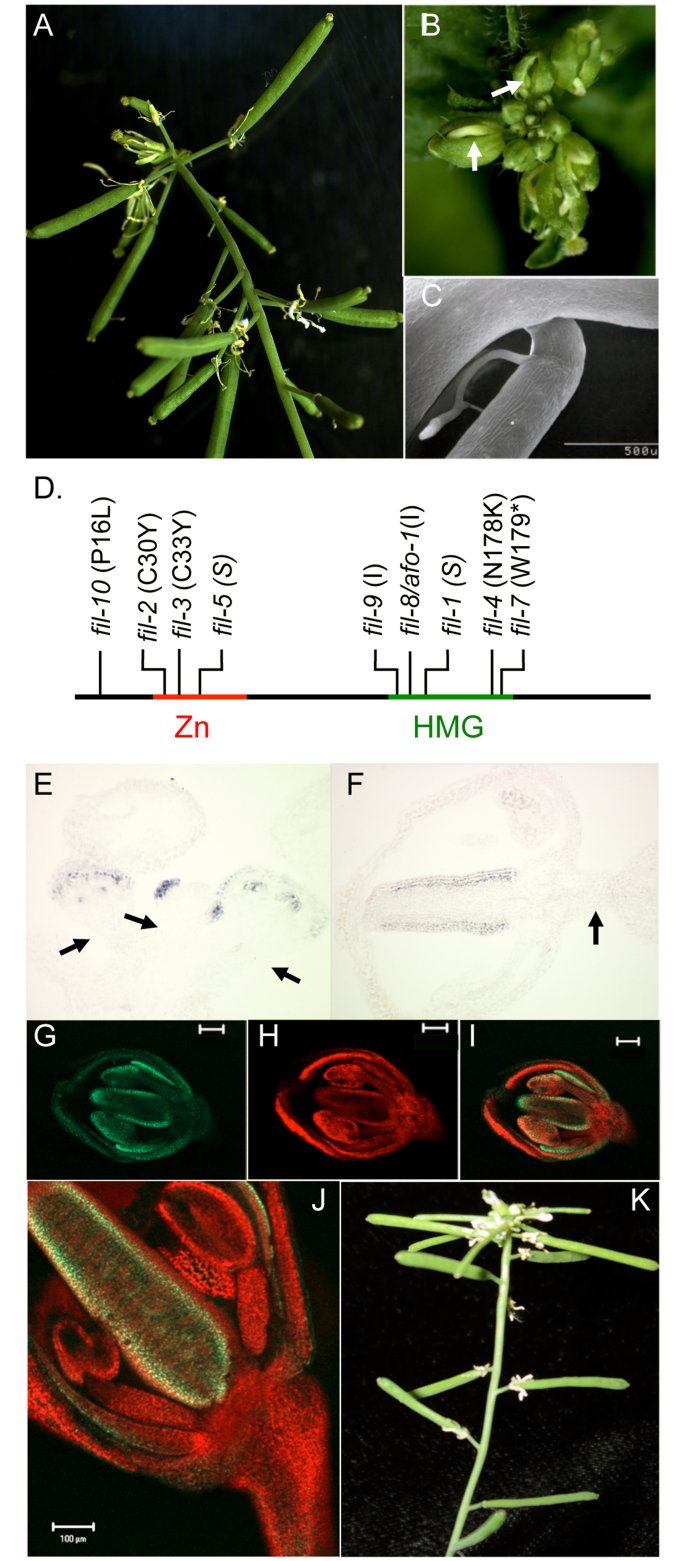
Mutations in *FIL* and *LAS* affect inflorescence architecture in a similar fashion. (A-C) *bp er fil-4* plants showing elongated pedicels (A), upward-oriented floral buds with gaps between sepals (arrow; B) and bends in pedicels at filamentous organs (C). (D) Locations of characterized mutations in the *FIL* gene. The nature of each mutation is shown in parentheses: I = insertional mutant, S = splice junction mutant; the asterisk represents a stop codon. (E-F) *In situ* hybridization with a *FIL* probe showing expression in sepal primordia (central bud) and in floral organs of older, peripheral buds (E), and gynoecium valve expression in a stage 9 pedicel (F). Note the absence of *FIL* expression in pedicel tissue (arrows) at stages that precede the period of pedicel elongation [59]. (G-I) A collage of a stage 9 bud from a transgenic plant expressing a *FIL*pro::*FIL*::*GFP* transgene. The left panel shows FIL::GFP expression on the abaxial side of floral organs; the middle panel is the chlorophyll autofluorescence (red channel) and the right panel is the merged image. (J) Mature flower illustrating FIL::GFP in floral organs only. (K) The *bp er las-11* triple mutant exhibits a phenotype nearly identical to that of *bp er fil-10*.

The *fil-10* mutation also conditions floral morphological phenotypes when combined with *er*, but these are typically much less severe than those that have been reported for strong *fil* alleles [34–36,42]. *fil-10 er* floral meristems initiate normally and generally produce four symmetrically arranged sepals (Fig 3A) that do not elaborate bract-like organs. In accordance with the phenotype of older flowers, the margins of young sepals are often separated by gaps that expose inner whorl organs (Fig 3B). Partial sepal-to-carpel homeotic transformations occasionally manifest as stigmatic tissue formed at the tips of medial first whorl organs (Fig 3C). In other cases, first or third whorl organs develop as radial filaments (Fig 3D). In the fourth whorl, gynoecia are often crooked or bent (Fig 3E), likely due to contact of the gynoecium tip with the inner face of a sepal (Fig 3F) and protruding stylar tissue is observed on medial sides (Fig 3G). We also examined the effect of a stronger *fil* allele in the *bp er* background. While the *fil-4* allele suppresses *bp er* in a similar fashion to *fil-10* (Fig 4), *bp er fil-4* plants display more severe stem and floral phenotypes that include phyllotaxy defects, the reduced floral cluster bearing type B flowers, and in many instances floral organ identity is severely compromised, manifested as filamentous organs (see S2 Fig). These defects mimic those of strong *fil* alleles. In summary, broad morphological defects in *fil-10 er* flowers support others’ findings that *FIL* plays an important role as a general regulator of floral organogenesis [34–36, 42], but define *fil-10* as a weak allele that impinges upon both BP and ER signaling.

### *fil-10* does not influence floral meristem identity

Previously we demonstrated that reduced floral meristem identity in *leafy* (*lfy*) mutants suppresses *bp er* pedicel phenotypes [33]. Reduced floral fate results in increased numbers of axillary stems and less prominent receptacles. Unlike *lfy*, our observations indicate that suppression of *bp er* pedicel phenotypes in *fil-10* is not due to changes to floral identity. First, axillary branch number is similar between *bp fil-10 er* (1.9 ± 0.2) and *bp er* (2.1 ± 0.1). Second, *fil-10* and *fil-10 er* receptacles enlarge (Fig 1J), but this feature is compromised when lfy is also mutant (in *bp er lfy-5* [33]). Third, we crossed *bp er fil-10* to *ap1-1 er* to examine the effect of *fil-10* in another known floral identity mutant. Similar to the effect of *lfy-5*, *ap1-1* suppressed the *bp er* pedicel phenotypes, but we also observed a novel floral phenotype that is not present in *ap1-1* or *fil-10* plants. In *bp fil-10 ap1-1 er* and *fil-10 ap1-1 er* flowers, medial first whorl organs of all flowers displayed carpel-like features that included stigmatic tissue at tips and along margins, style-like tissue adjacent to margins, ovules along margins and an overall hooded morphology (Fig 3H). Importantly, secondary flowers evident in axils of first-whorl organs in *ap1-1* were never observed in *fil-10* backgrounds, suggesting that *fil-10* flowers are fully determinate. Collectively, these results indicate that *fil-10* does not compromise floral identity as is the case for stronger *fil* alleles [34, 36], (and S2 Fig). Thus, *FIL* may interact with *BP* and *ER* to influence floral architecture and pedicel growth downstream of floral meristem fate specification.

### *fil-10* does not impact pedicel development through its effect on organ polarity

It is well established that FIL contributes to the emergence of organ polarity by specifying abaxial identity of lateral organs [35]. To determine whether a reduction in abaxial organ identity contributes to suppression in *bp er fil-10*, we crossed *bp er* with *kanadi-1* and *kanadi-2*, which show abaxial-to-adaxial transformations in leaves and floral organs [38, 52–56]. We saw no evidence of suppression of *bp er* pedicel phenotypes in *bp-4 kan1-2 er*, *bp-4 kan2-1 er* or *bp-4 kan1-2 kan2-1/+ er*, suggesting that lateral organ polarity *per se* does not significantly influence pedicel morphology. Because the *KAN* genes are expressed in stem tissue where they play a role in vascular patterning [55] we also tested the relationship between organ polarity and pedicel development by removing the function of *ASYMMETRIC LEAVES2* (*AS2*) from *bp er fil-10* plants. KAN exerts its function in part by repressing *AS2* [57], an adaxial regulator that is expressed in leaves and floral organs but not in internodes or pedicels [25, 58]. Because removal of *AS2* from an *er* background increases abaxial fate in lateral organs [58], we reasoned that this could counteract the loss of abaxial identity due to the *fil-10* mutation, phenocopying the *bp er* pedicel phenotypes. However, although quadruple *bp er fil-10 as2-101* mutants gave rise to shorter pedicels, removal of *AS2* did not affect pedicel angle (Table 1), consistent with the *kan* data suggesting that organ polarity does not significantly impact pedicel morphology.

**Table 1:** The influence *AS2* on pedicel architecture.

Genotype^a^	Pedicel Length (mm)	Pedicel Angle (degrees)^b^
*bp er fil-10*	2.75 ± 0.05	93.1 ± 0.9
*bp er fil-10 as2-101*	1.75 ± 0.09	95.9 ± 1.3

^a^For *bp er fil-10*, n = 189. For *bp er fil-10 as2-101*, n = 55.

^b^Angle between the inflorescence axis and the adaxial face of the pedicel.

Pairwise T-tests revealed that the change in pedicel length is statistically significant (p<0.005), while the change in pedicel angle is not (p = 0.34).

### Identification and molecular characterization of *fil-10*

The original *bp er* suppressor mutation (termed *sup2*) was mapped to a 660kbp region on chromosome 2 between the T8M12 and GBF3 markers. Scanning annotation units in this chromosomal region showed that the *YABBY* gene *FILAMENTOUS FLOWER* (*FIL*) is located approximately halfway between the two markers. Similarities between *fil* and *sup2* phenotypes, including compromised fecundity, filamentous organs, and style defects prompted us to test whether other *fil* alleles could suppress *bp er*. Crossing the intermediate *fil-4* allele into *bp er* produced plants with elongated pedicels, although pedicels often bend down at filamentous structures formed on abaxial sides (Fig 4A–4C). We next crossed *bp er fil-4* with *bp er sup2* in a complementation test. Progeny plants exhibited a suppressed *bp er* phenotype, indicating that the lines contain mutations in the same gene. To confirm that *FIL* is mutated in *sup2*, *FIL* cDNA and genomic fragments isolated from *bp er sup-2* plants were cloned and sequenced, revealing a P16L mutation located upstream from the Zn finger domain (Fig 4D). Taken together, these experiments indicate that the *sup2* phenotype is due to a mutation in the *FIL* gene and we propose *fil-10* as the allele designator.

*FIL* is expressed in leaves and floral organs and acts to specify abaxial organ fates and promote blade outgrown, in part by repressing *KNOX1* genes [32]. In addition, the finding that *fil* mutations suppress the *bp er* phenotype suggested that in this background, *FIL* might be ectopically expressed in pedicels to modulate their development. However, *in situ* hybridization with a *FIL* probe failed to detect *FIL* transcripts in *bp er* pedicel or internode tissue at all floral stages tested (Fig 4E and 4F), suggesting that *FIL* may function non-cell-autonomously from flowers to impact pedicel development. To more specifically test this hypothesis at the protein level, we constructed a *FILpro*::*FIL*::*GFP* transgene and generated transgenic lines in both wildtype and *bp er* plants. Examination of young buds revealed the characteristic abaxial domain expression of *FIL*, but in no case, at any stage of floral development, did we observe GFP fluorescence in developing pedicels (Fig 4G–4J). Moreover, pedicel angle defects begin to be manifest after about stage 11 of floral development [33], and the bulk of pedicel elongation also takes place after stage 11 [59], suggesting that pedicel development is spatially (and temporally) separated from FIL expression domains in floral organs. Finally, the introgression of the *lateral suppressor* (*las-11*) mutant into *bp er* confers a phenotype that is nearly identical to that of *bp er fil-10* (Fig 4K). Recognizing that *LAS* regulates axillary meristem activity [60], and has been implicated in transducing the *FIL* non-cell-autonomous signal from peripheral domains of the meristem to the CZ [39], we reason that FIL’s effect on stem and pedicel development is likely mediated in a similar fashion. That the origin of the signal is superior to the pedicel is inferred by amelioration of the stripes of undifferentiated abaxial tissue that originate and are broadest at the receptacle in *bp er*, and trace the path of the vasculature down the inflorescence stem [15, 33], but which are suppressed in *bp er fil* mutants.

### *LEUNIG* and *YAB3* mutations differentially suppress the *bp er* phenotype

**Fig 5:**
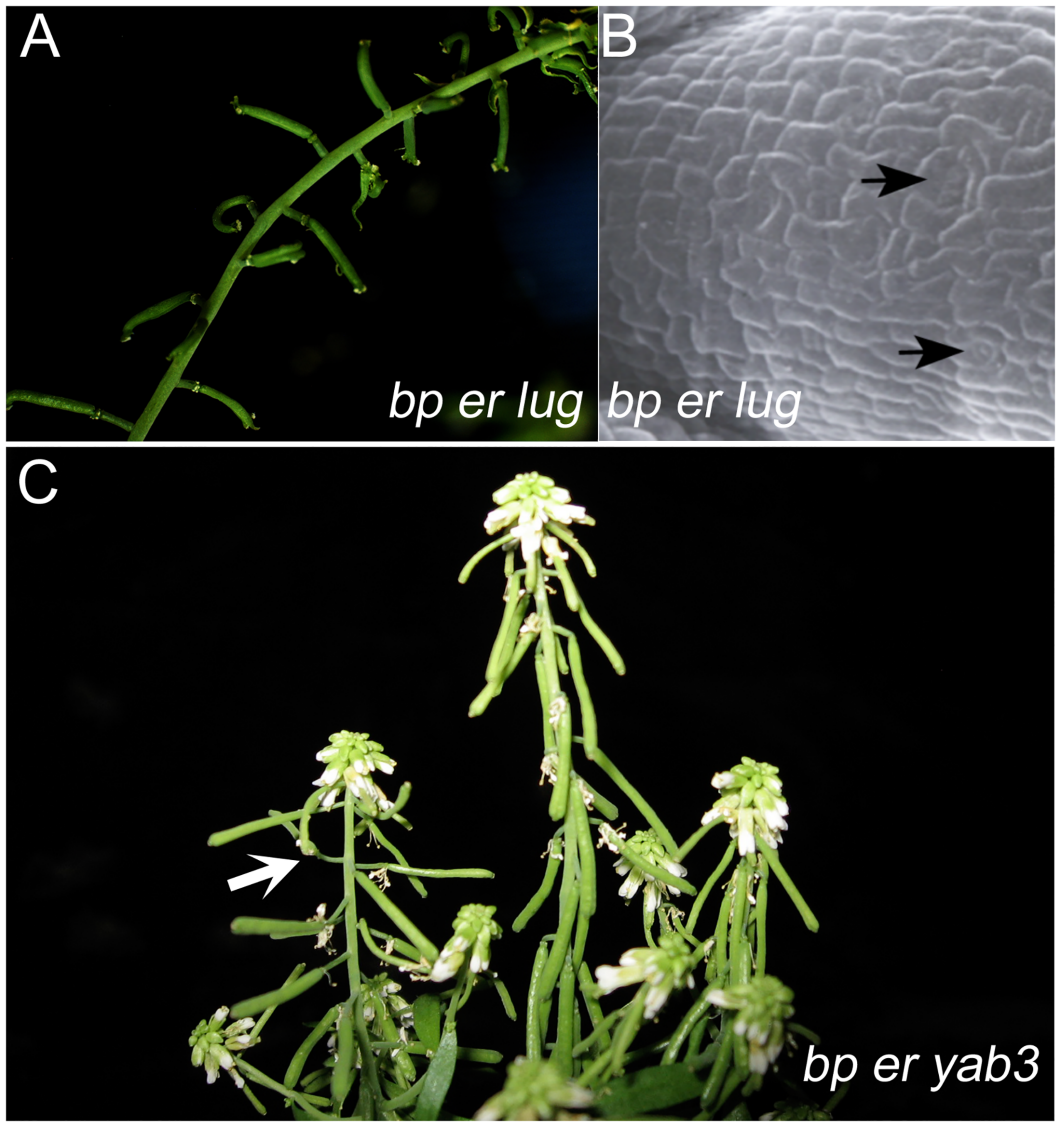
Suppressive effects of mutations in *leunig* and *yabby3*. (A) *bp er lug* plant showing suppressed pedicel angles. (B) *bp er lug* abaxial pedicel showing enlarged cells and stomata (arrows). (C) *bp er yab-3* plant. In rare cases, we observed pedicel suppression effects (arrow) of some axillary branches on plants which otherwise exhibited the *bp er*-like habit.

YABBY proteins are known to form complexes with Gro/Tup1 co-repressors such as LEUNIG (LUG) [40]. *LUG* is ubiquitously expressed and *lug* mutants show homeotic transformations in the flower [61]. In addition, LUG and its interacting partner protein SEUSS (SEU) act to control organ polarity and other aspects of plant development [62–64]. Upon crossing *bp er* and *lug*, we found that *bp er lug-1* plants also exhibited suppressed pedicel phenotypes (Table 2) wherein pedicels are elaborated perpendicular to the stem axis and elongate to some extent (Fig 5A). The stomata-free stripe of cells on the abaxial side of *bp er* pedicels is also ameliorated, giving rise to normal epidermal patterning that includes stomatal development (Fig 5B).

**Table 2:** Effects of BP and LUG on pedicel morphology.

Genotype	Pedicel Length (mm)^a^	Pedicel Angle (degrees)^a^^,^^b^
L*er*	3.7 ± 0.1	55 ± 2
*lug-1 er*	2.8 ± 0.2	48 ± 7
*bp er*	0.41 ± 0.03	143 ± 2
*bp er lug-1*	0.99 ± 0.07*	84 ± 3*

^a^ n>20. Each value represents the mean ± standard error.

^b^Angle between the inflorescence axis and the adaxial face of the pedicel.

* Pairwise T-tests reveal significant differences for both pedicel length and angle for *bp er* vs *bp er lug-1* (p<0.05).

Given that some *YABBY* proteins are expressed in overlapping domains, interact physically with one another, and can rescue mutations in other *YAB* genes [40, 65, 66], we reasoned that mutations in *YAB3*, a close *FIL* relative, also might be able to suppress the *bp er* phenotype. We generated the *bp er yab3* triple mutant but found that *yab3* was ineffective in suppressing the *bp er* phenotype (Fig 5C). In very rare instances, secondary branches displayed some degree of suppression on plants that were otherwise *bp er*-like. Thus, the *fil-10* suppression phenomenon generally cannot be phenocopied by *yab3*, although the two genes are functionally redundant in other contexts (e.g. vegetative development; [32, 35]).

### Transcription profiling reveals possible mechanisms of FIL action

Our previous studies provided evidence for the existence of a vascular-borne signaling molecule whose synthesis, activation, or trafficking influences inflorescence architecture [33]. We therefore undertook transcription profiling experiments with *bp er* and *bp er fil-10* inflorescences as a strategy to identify genes whose regulation is governed by FIL, anticipating that the identities of putative targets might suggest the nature of this signaling pathway. Triplicate samples of inflorescence RNA from the two genotypes were analyzed, and genes that exhibited more than a 2.5 fold change were functionally classified using both MapMan and Gene Ontogeny (GO) algorithms (S2/S3 Tables). The two lists are referred to hereafter as the UP list (genes upregulated in *bp er fil-10*, implying that FIL directly or indirectly represses these genes in *bp er*) and the DOWN list (genes downregulated in *bp er fil-10*, implying that FIL directly or indirectly activates these genes in *bp er*).

The UP list contains 71 genes. By normalizing these genes to their frequency in the functional classification groups, only the genes involved in RNA metabolism/transcription factor activity are over represented (p-value = 5.673^−4^). Twelve genes encode validated or putative transcription factors, including four Zn finger proteins, three AP2/EREBP domain factors, two homeobox domain proteins, one B3 domain protein, 1 JUMONJI family member, and one GeBP domain protein. A second category is a group of genes whose products are involved in regulated proteolysis. Lastly, there are 25 genes that encode products of unknown function, but in general there are no obvious patterns that implicate specific signaling pathways or other commonalities that inform how FIL executes its function. Rather, it appears likely that FIL may act in numerous processes by regulating a group of subordinate transcription factors.

**Fig 6:**
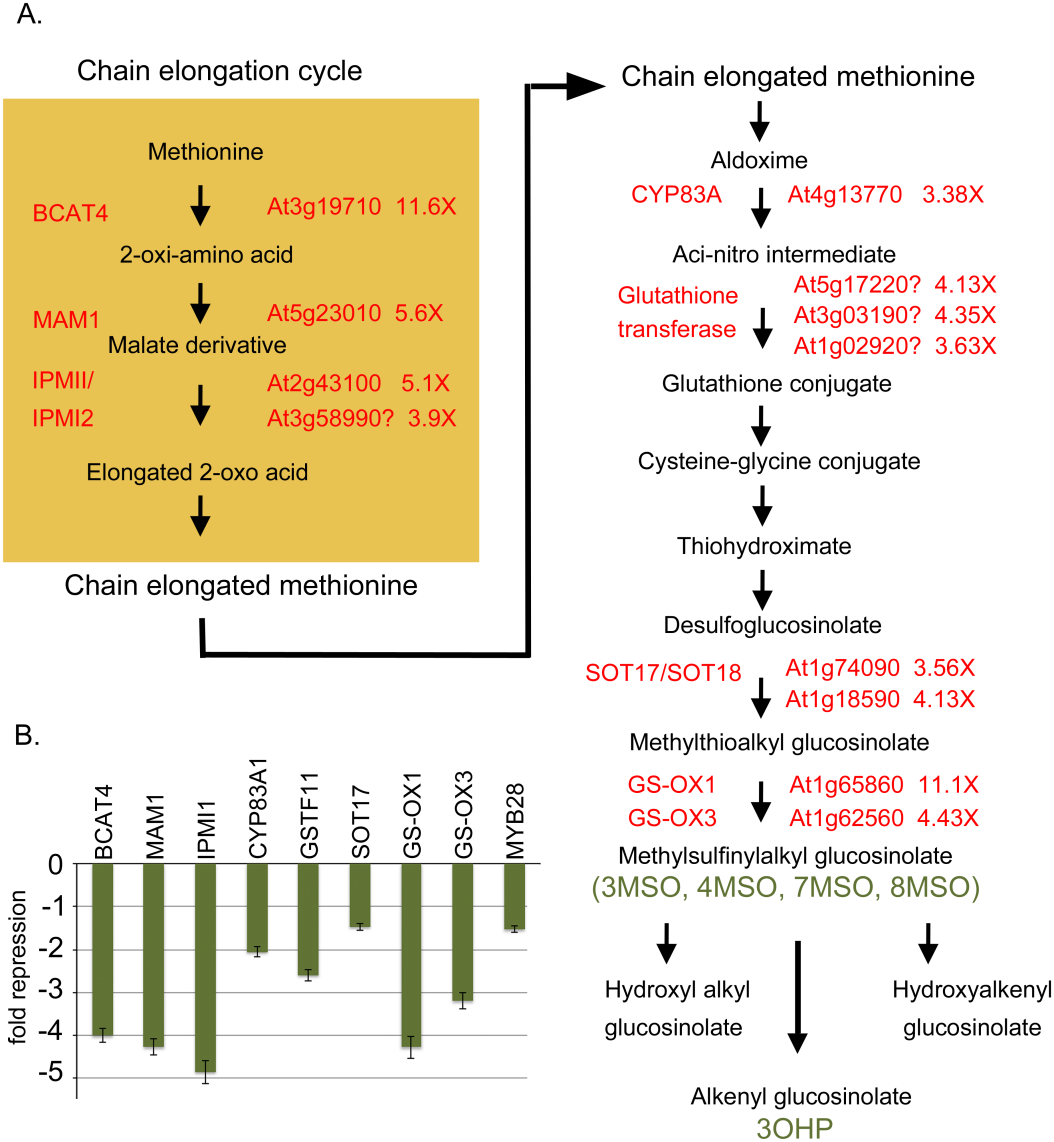
Aliphatic glucosinolate biosynthesis genes are down-regulated by *fil10*. (A) Schematic representation of the aliphatic glucosinolate biosynthetic pathway showing genes involved in various steps. The numbers beside the AGI identifiers indicate the change in expression of these genes in *bp er fil-10* suppressor vs. the parent *bp er* line as gauged by microarray analysis. Question marks indicate uncertainly about the involvement of these genes in the indicated steps. The green text identifies specific glucosinolate metabolites that are products of the enzymatic steps and for which quantitative analysis was performed (see Table 3). (B) QRT-PCR analyses of selected GSL biosynthetic genes, confirming down regulation of these genes in *bp er fil-10* verses the *bp er* parent. The *GSTF11* gene (At3g03190) was selected for analysis as its expression pattern is very similar to that of *FIL* (eFP browser data) and the gene has been implicated in GSL biosynthesis. The relative expression ratio of the *bp er fil-10* mutant is shown and error bars are the standard error of the mean. Pair-wise t-tests on linear transformed ΔCT values revealed that all differences are statistically significant (p<0.034).

The DOWN list of 63 genes was parsed into several categories that are statistically overrepresented. Trends are observed for members of the miscellaneous and secondary metabolism category, and normalization to the reference set of all genes reveals these classes are overrepresented by 7 and 18 fold (p-values are 4.82 x 10^−13^ and 3.4 x 10^−15^, respectively). Ten of the secondary metabolism genes are known to be involved in the synthesis or modification of glucosinolates (GSLs) and an additional four are suspected to play roles in GSL metabolism based on the biochemical steps involved and the predicted enzymatic function (e.g. glutathione transferases). In a similar vein, several of the transcription factors on the UP list belong to families whose members are known to be physically associated with GSL gene promoters to modulate their expression [67]. Fig 6 shows a schematic of the aliphatic glucosinolate biosynthetic pathway, overlaid with genes whose expression is down regulated in *bp er fil-10*. Glucosinolate biosynthesis is initiated from tryptophan, phenylalanine, methionine or chain-elongated methionine derivatives [68]. The chain elongation cycle involves MAM1, IPMI isozymes, and BCAT4, whose collective function is to extend the amino acid derived substrates. These products feed into the central pathway that utilizes several cytochrome P450 monooxygenases, glutathione addition, and sulfotransferase and oxygenase activities to generate methylsulfinylalkyl glucosinolates. To validate the transcription profiling results we conducted QRT-PCR on several of the targets: *MAM1*, *BCAT4*, *IPMI1*, *CYP83A1*, *SOT17*, *GS-OX1*, *GS-OX3* and *GSTF11* (Fig 6B). QRT-PCR experiments revealed that all of the target genes tested are indeed downregulated in the *fil-10* background, though generally not to the extent reported by the microarray analyses. In addition, the *MYB28* gene, a known activator of aliphatic glucosinolate biosynthesis is also downregulated [69, 70]. Control of *MYB28* by FIL may explain the wide-ranging changes in GSL gene expression.

**Fig 7:**
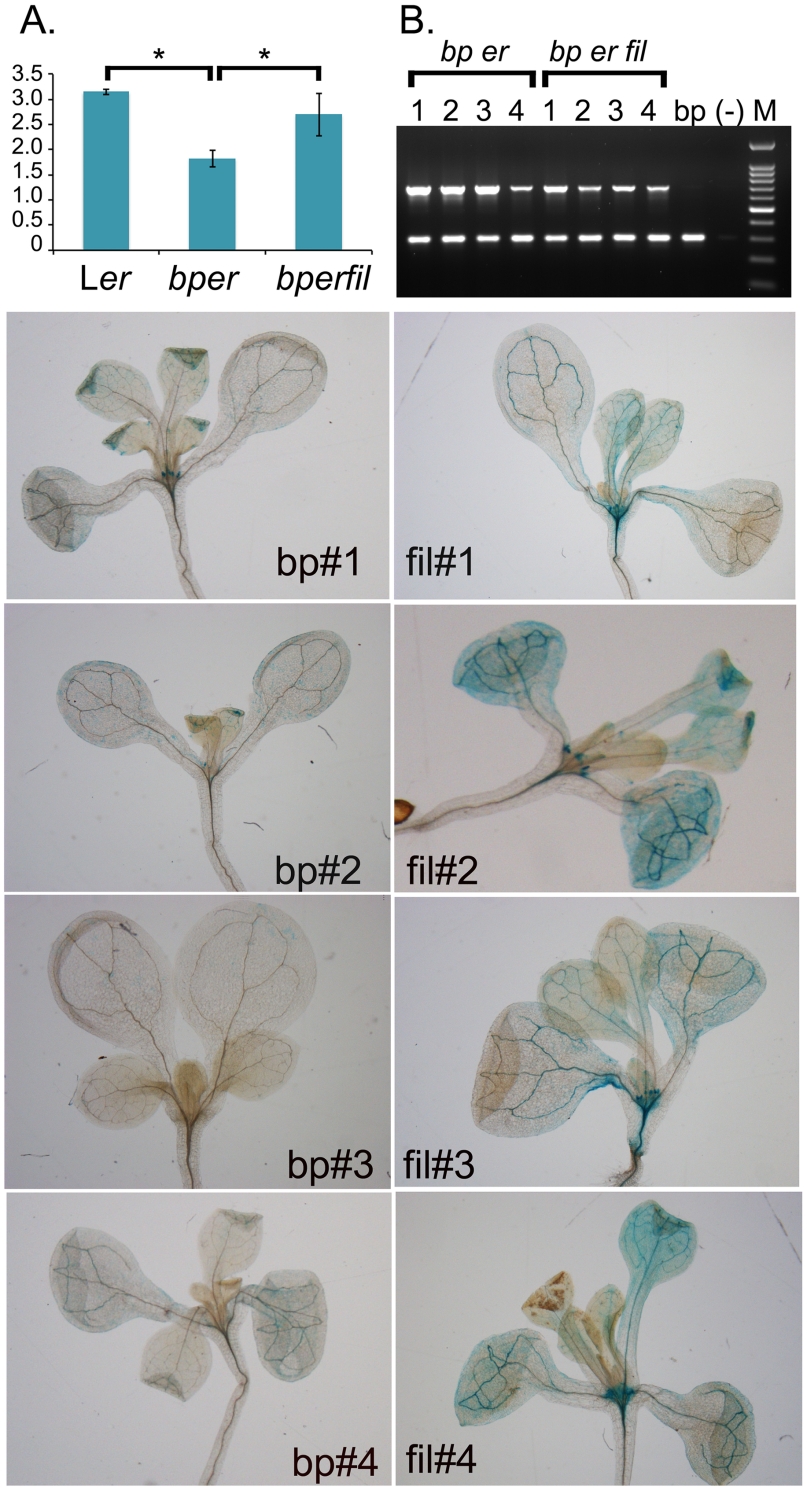
Auxin levels are altered in *bp* and *fil* mutants. (A) Auxin levels in L*er*, *bp er* and *bp er fil-10*. Wildtype *FIL* is required for the *bp er* phenotype and is associated with lower auxin levels. Pairwise T-tests revealed significant differences between L*er* and *bp er* (p < 0.001), and between *bp er* and *bp er fil-10* (p = 0.01). (B) Multiplex PCR on four independent transformants of both *bp er* or *bp er fil-10* harboring the auxin reporter DR5::GUS. The lower band represents a single copy control gene (AMI) while the upper band assesses the presence/level of the DR5::GUS reporter gene. The *bp* lane is a non-transformed control, (-) is no DNA template. Lower left panels: X-gluc stained seedlings of four independent *bp er* transformants. Lower right panels: X-gluc stained seedlings of four independent *bp er fil-10* transformants. In all cases, the *bp er fil-10* suppressor lines exhibited broader and more intense staining than the *bp er* lines, despite the fact that the copy number of the auxin reporter gene was similar or even lower in the *bp er fil-10* lines (panel B).

Downregulation of GSL biosynthetic genes led us to hypothesize that there is an altered glucosinolate metabolite pool in *bp er fil-10* plants. To assess this, we conducted glucosinolate profiling on the single L*er* mutant, the *bp er* double mutant and the *bp er fil-10* suppressor. For many of the metabolites measured, mutations in either *bp* or *fil* led to significant changes in GSL metabolite levels (Table 3 and S2 Fig). The levels of several GSLs, including 3OHP, 4OHB, 4MSO, 7MSO, and 8MSO, were altered in the *fil* mutants in comparison to *bp er* but these were not consistent between *fil-4* and *fil-10* suggesting that, unlike the suppression phenotype, the GSL profiles are allele specific. Interestingly, the level of 3-indolyl methylglucosinolate (I3M) is elevated in both suppressor lines relative to the *bp er* parent, and this phenotype could be linked to the suppression ability of these alleles (S2 Fig). Indolic glucosinolates are derived from tryptophan, which also contributes the indole ring to auxins such as IAA. Given that mutations in several genes encoding enzymes involved in both aliphatic and aromatic GSL synthesis impact auxin metabolism [71–80], we reasoned that auxin levels might be altered in these plants. To investigate this hypothesis, we examined IAA levels in inflorescences from the three genotypes (Fig 7A). L*er* inflorescences contain on average about 3ng/g FW of IAA. The dwarf-like double mutant *bp er* has lower levels of IAA (40% of L*er* level), which may contribute to its diminutive stature. The *bp er fil-10* suppressor line essentially restores IAA levels to that of L*er*, and we postulate that elevated auxin levels are in part responsible for more robust growth of the suppressor line. The enhancement of auxin levels is corroborated by examining independent DR5::GUS transformants of *bp er* and *bp er fil-10*. In the *bp er* background, DR5::GUS signals mimic the wildtype pattern for auxin maxima [81], showing staining foci at leaf tips, hydathodes, young leaf primordial/stipules, root tips, and vascular tissues. In the *bp er fil-10* suppressor background, the qualitative GUS staining pattern is mostly unchanged, but intensity is greater in all cases. This is particularly evident at the shoot apex and within the vascular tissues, and in most transformants, numerous cells within the leaf blade also display staining.

**Table 3:** Glucosinolate metabolites in L*er*, *bp er* and *bp er fil-10*.

Genotype/	3OHP^1^	4OHB	3MSO	4MSO	7MSO	8MSO	I3M	4OHI3M	NMO
L*er*	5.90±0.67	0.165 ± 0.02	0.069 ± 0.013	0.032 ± 0.003	0.113 ± 0.016	1.24 ± 0.18	0.16 ± 0.016	0.014 ± 0.004	0.022 ± 0.005
*bper*	6.0 ± 0.76	0.200 ± 0.04	0.123 ± 0.026	0.030 ± 0.003	0.135 ± 0.021	1.38 ± 0.29	0.08 ± 0.015	0.011 ± 0.004	0.013 ± 0.005
*bperfil*	4.59 ± 0.67	0.11 ± 0.03	0.179 ± 0.078	0.026 ± 0.01	0.106 ± 0.019	0.81 ± 0.10	0.09 ± 0.019	0.006 ± 0.004	0.012 ± 0.003
T-tests^2^									
L*er* vs. *bper*	0.7948348	0.05654988	0.000333266	0.241202909	0.058579013	0.2900458	4.31E-07	0.183791614	0.00650812
*bper* vs *bperfil*	0.0294717	0.00058607	0.100847632	0.406288507	0.029228242	0.0007679	0.0906205	0.097505342	0.787914392

^1^Abbreviations: 3OHP: 3-hydroxypropyl; 4OHB: 4-hydroxybutyl; 3MSO: 3-methysulfinyloctyl; 4MSO: 4-methysulfinyloctyl; 7MSO: 7- methysulfinyloctyl; 8MSO: 8-methysulfinyloctyl; I3M: indol-3-ylmethyl; 4OHI3M: 4-hydroxy-indol-3-ylmethyl; NMO: N-methoxy-indol-3-ylmethylglucosinolate.

^2^Student’s T-test was carried out for the pairwise comparisons of L*er* vs *bp er* and *bp er* vs *bp er fil*. P-values are shown; confidence intervals of p< 0.05 are highlighted

**Fig 8:**
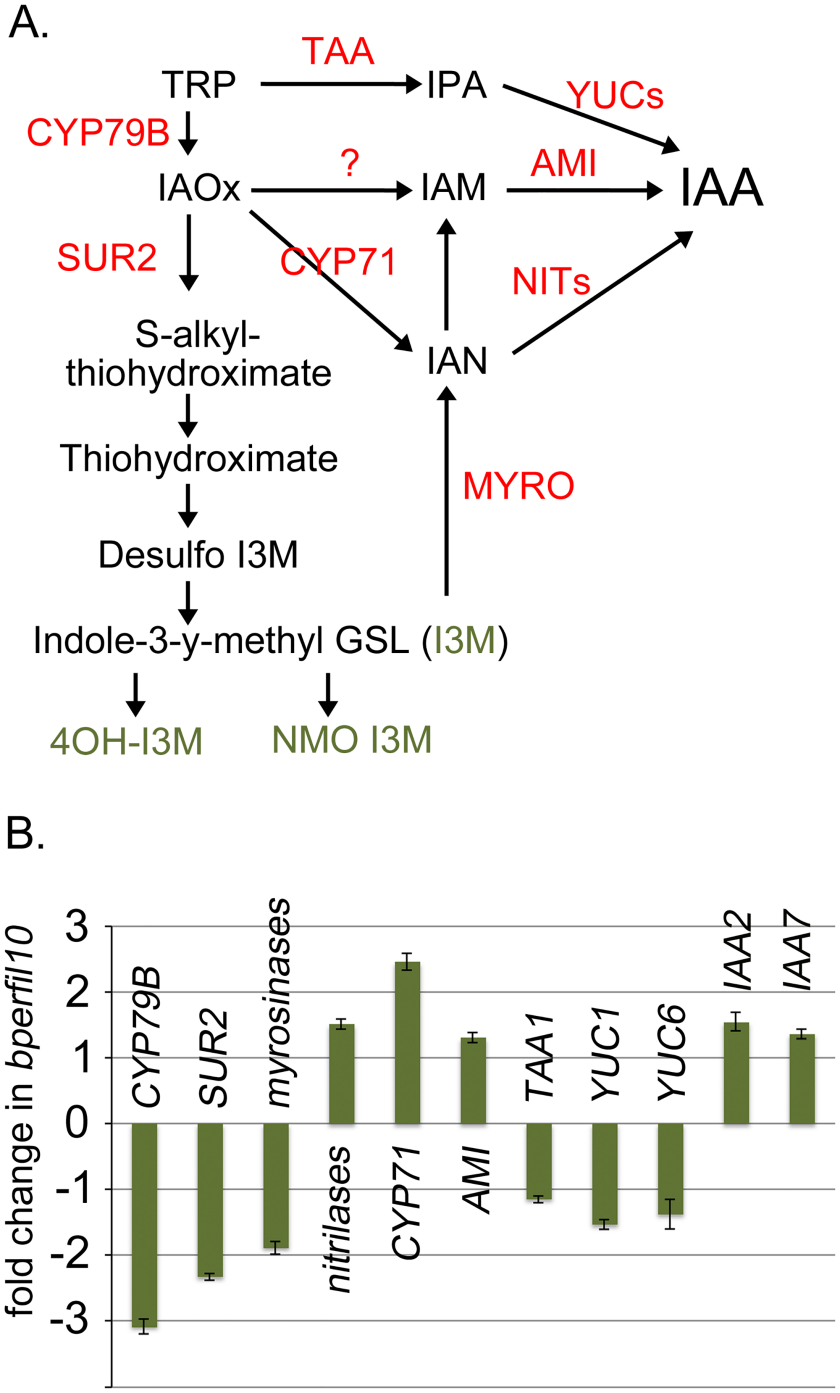
Changes in the expression of indolic glucosinolate and auxin biosynthesis genes in *bp er fil-10*. (A) Inferred and speculative intersections of auxin and glucosinolate biosynthetic pathways. Some pathway steps are embellished with gene designations where empirical data implicate specific associations (red text). The green text identifies specific glucosinolate metabolites that are products of the enzymatic steps and for which quantitative analysis was performed (see Table 3). (B) QRT-PCR data on selected genes implicated in indolic glucosinolate and auxin metabolism. The relative level of transcripts in *bp er fil-10* vs *bp er* is shown. Error bars represent standard error of the mean. Pairwise t-tests on linear transformed ΔCt revealed all differences to be statistically significant (p<0.02) except *YUC1* (p = 0.069) and *YUC6* (p = 0.55).

Despite a wealth of data on GSL biosynthetic mutants that influence auxin levels, the mechanistic connection between GSL biosynthesis and IAA production has not been elucidated. However, an aromatic pathway intermediate, IAOx, can be converted to IAA by reactions involving the intermediates IAN or IAM (reviewed in [82–83]), and in addition, IAA can be produced indirectly through GSL degradation by myrosinases (Fig 8A). To investigate these possibilities we conducted QRT-PCR on genes involved in indolic GSL biosynthesis and IAA biosynthesis. In general, the expression of most of these genes was either downregulated or unchanged, but changes in the expression of several genes are intriguing. First, direct IAA production through TAA and the YUCCA enzymes is likely reduced as *TAA1*, *YUC1*, and *YUC6* were found to be downregulated in *bp er fil-10* (Fig 8B). Importantly, the expression of CYP71A13 and an indole-3-actamide hydrolase (AMI1) are upregulated, which may provide a shunt to partition GSL metabolites into auxin biosynthesis. In addition, elevated expression of nitrilases may also convert IAN to IAA, though in an independent experiment, the nitrilases were found to be downregulated (see S1 Fig). As similar trends were observed for the other genes investigated, it is unclear why the nitrilases displayed this variation. QRT-PCR analysis of these genes in the *bp er fil-4* background revealed higher levels of myrosinase mRNA, which may contribute to shunting indole-3-glucosinolate into the auxin biosynthesis pathway (S2 Fig). In addition, elevated levels of CYP71A13 mRNA may also contribute to conversion of GSL metabolites to IAA. We infer that in inflorescences, both the *fil-4* and *fil-10 suppressors* orchestrate changes in the levels and shuttling of GLS metabolites that influence local auxin concentrations, conditioning changes in gene expression that affect shoot architecture. It is also likely that some of the uncharacterized genes, and/or those encoding enzymatic functions implicated in metabolite interconversions (e.g. cytochrome P450s) may provide a heretofore unrecognized means to alter auxin biology.

## Discussion

Distinct plant species elaborate organs in genetically defined patterns, giving rise to the species’ characteristic inflorescence architecture and general plant habit. Mutant screens have identified a large number of genes that influence aspects of meristem specification and maintenance, boundary formation, phyllotaxy, organ identity, and hormone synthesis, transport and perception (reviewed in [84]). Class 1 *KNOX* genes play integral roles in many of these processes, and their expression is subject to activation or repression, spatially and/or temporally, by several well-characterized factors and auxin [3]. In general, the KNOX1 proteins condition a replication competent state and prevent differentiation in the meristem, and their expression is downregulated as cells are recruited into lateral organ primorida, yet other studies have revealed that reactivation of *KNOX* genes occurs in leaves of compound leaf species [23]. One class of negative regulators is the *YABBY* family of transcriptional repressors, which play roles in SAM activity, floral development, leaf lamina growth control, promotion of abaxial cell fate, and inflorescence phyllotaxy [34–36, 42].

Our studies reveal that mutant alleles of the *YABBY* family member *FILAMENTOUS FLOWER* suppress many of the developmental aberrations conditioned by the class 1 *KNOX* gene *brevipedicellus*. Previous studies have implicated FIL in downregulating *KNOX* genes in lateral organ primordia, and in higher order *fil/yab* mutants, ectopic meristem formation in leaves may be due to KNOX gene derepression [32]. It is thus possible that *fil-10* mediated suppression of *bp er* phenotypes might be related to changes in the expression of other *KNOX* genes. In this regard, while our microarray data indicated that *KNAT2* is upregulated by nearly 3 fold, QRT-PCR experiments revealed that the magnitude of this change is only 1.4 fold (S3 Fig). Moreover, the expression of *KNAT6* and *STM*, known modulators of meristematic activity, are unchanged [85–88]. It therefore seems unlikely that *KNOX* gene reactivation plays a prominent role in rescuing the *bp er* phenotype. In all likelihood, the large number of genes that are affected by the *fil-10* mutation, which includes more than twelve transcription factors, specify a complex network affecting numerous cellular processes that will be difficult to dissect. Two of these genes encode proteins with sequence similarity to the PLETHORA family that regulates inflorescence phyllotaxy by modulating local PIN1 activity [89], and our analyses of auxin in the *bp er* and the *fil-10* suppressor lines, together with the phenotypic alterations they display, are consistent with localized changes to growth regulating molecules.

### FIL acts non-cell autonomously to modulate development

FIL contributes to several aspects of inflorescence architecture. In vegetative development, *FIL* is expressed in young leaf primordia, along the abaxial sides of leaves, and in the peripheral zone of the SAM [34–36]. During early floral development, *FIL* expression is confined to cryptic bracts/sepals and later is found on abaxial sides of floral organs [35, 39]. Finally, during fruit development *FIL* is expressed in valve and presumptive valve margin cells where it contributes to the activation of genes required for valve margin development [35, 90]. In both developing leaves and fruit, FIL influences tissue identity in part by repressing *KNOX* genes, but apparently does so in a non-cell autonomous fashion. In leaf primordia, interruption of peripheral *YAB1 (FIL or FIL/YAB3*) expression alters meristem central zone activity to produce phyllotaxy defects, and in situ hybridization and reporter gene activities indicate that *FIL* is not expressed in the affected domains [39]. A suppressor screen identified LATERAL SUPPRESSOR (LAS) as a transducer of this mobile signal. Our introgression of the *las-11* mutation into the *bp er* background resulted in architectural changes to plants that generally mimic the *bp er fil* phenotypes. Together with the in situ hybridization and FILpro::FIL::GFP reporter expression patterns (Fig 4), this observation indicates that the non-cell autonomous signalling that operates between PZ/CZ in leaf development is also employed to regulate pedicel and internode elongation and patterning. Finally, this regulatory module likely is key to repressing *BP* in the replum during fruit development. In *fil* and *fil/yab3* mutant backgrounds, *BP* expression is enhanced in replum tissues, which are larger and differentiate stomata [91], a phenotype that is similar to stripe suppression and stomatal differentiation in *bp er fil-10* pedicels (Fig 1). In fruits, the non overlapping expression patterns of medial (BP) and lateral (FIL) factors support the contention that FIL signals non autonomously from the adjacent lateral tissue to the medial (replum) tissue to influence replum morphogenesis [91]. Whether LAS is involved in this context is unknown, but it is clear that FIL employs one or more mobile signals to dictate multiple aspects of plant development in Arabidopsis.

### Changes in auxin and glucosinolate profiles modulates meristem activity

*BP* expression is linked to auxin metabolism, as exemplified by its ectopic expression in leaves of *axr1* and *pin1* mutants, and in leaves of plants treated with auxin transport inhibitors [92]. Such studies implicate auxin as a negative regulator of some *KNOX1* genes, possibly acting through ARF6/ARF8 [93]. Conversely, chromatin immunoprecipitation of maize *KNOTTED1* target loci, coupled with RNAseq, revealed that genes involved in auxin biosynthesis, transport and signaling are upregulated in dominant *Kn1-N* mutants [94]. Although we have not performed similar studies on *bp* mutant plants, we found a reciprocal relationship in which loss of *KNOX1* (*bp*) function is correlated with reduced IAA levels in inflorescences (Figs 7 and 8). This in turn is associated with reductions in internode and pedicel elongation, and other developmental/tissue identity phenotypes. These data are consistent with the existence of a negative regulatory loop by which *KNOX1* genes could attenuate their own expression by enhancing auxin biosynthesis, transport and/or signaling.

Auxin is implicated in many facets of plant development and in responses to external stimuli. We propose that changes in auxin levels underpin the growth habit differences between *bp er* and the *bp er fil* suppressor lines. There are numerous literature reports that support this contention. For example, in *arf6/arf8* auxin response mutants of both Arabidopsis and tomato, internode and/or floral organ elongation is compromised [93,95]. Second, in *crm/big/tir3* mutants that exhibit shortened internodes and pedicels, the basis of this defect is linked to aberrant polar auxin transport [96–99]. Indeed DR5 reporter signals in *crm1-1 and big-j588* mutants is very much attenuated relative to wildtype [98, 100], suggesting lower auxin levels in this background, and *pCYCB1;1*::*CYCB1;1-GUS* signals were also reduced [99], implying that one role of CRM/BIG/TIR3 is to promote cell division. These authors also conducted morphometric analyses of well characterized auxin signaling mutants, *axr1-12*, *arf1-3 arf2-6*, and *nph4-1 arf19-1*, and showed that in all cases, shorter pedicels and internodes are due to defects in both cell size and cell number [99]. We previously reported that *bp* conditions similar cellular and tissue defects versus the L*er* parent line [15], and herein we demonstrate that auxin levels in seedlings and/or inflorescences are significantly lower in *bp er* than in either L*er* or *bp er fil-10*. Taken together, the data support the hypothesis that lower auxin levels are related to the stunted growth of *bp er* plants and that the molecular mechanisms that restore auxin levels serve to promote more robust growth in *bp er fil-10* plants.

A remaining question is how might *fil-10* influence auxin levels? The microarray data revealed no substantial changes in known auxin biosynthetic genes and QRT-PCR experiments indicate that the auxin-related genes tested (TAA, YUC1, YUC6, which in wildtype are most highly expressed at the shoot apex and/or in young floral buds [101]) are significantly downregulated. Although other pathways exist to synthesize IAA [82,83] the microarray data implicated downregulation of MYB28 and altered regulation of a number of glucosinolate metabolism genes as potentially creating a metabolic shunt from GSL pathways into those that produce IAA. MYB28 is part of a group of R2R3 MYB genes that activates aliphatic GSL biosynthetic genes [68–70, 102]. Loss and gain-of-function studies of MYB28 reveal that perturbing GLS can give rise to developmental defects and uncovers a reciprocal relationship between aliphatic glucosinolates and indolic glucosinolates (particularly I3M; [68–70]. Interestingly, mutant analysis of other GSL biosynthetic genes also reveals crosstalk between the aliphatic and indolic pathways [66, 75, 103, 104], but the intersections of these two pathways are not entirely clear. It is possible that the enzymes involved could utilize both aliphatic and indolic substrates, but the enzymology data is sparse (e.g. there is a 50 fold higher affinity for IAoX by CYP83B, vs CYP83A, [72]). Thus, while pathway intermediates have been determined by feeding experiments, the flux of metabolites is not linear and in all likelihood there are multiple points where shunts and feedback steps exist.

Although MYB28 is downregulated in *fil-10*, the GSL biosynthetic genes that are affected are only a subset (three of ten affected genes) of those known to be altered by 35S::MYB28 overexpression [70]. It is therefore likely that the influence of FIL on other genes is a major contributing factor to the elevated IAA levels we observe. In the *fil-10* suppressor, up-regulation of both *CYP71A13* [105] and *AMI* [106] could provide a means to channel IAoX to IAA, and in the *fil-4* suppressor, CYP71A13, AMI, and myrosinase activity could contribute to this shunt. Feeding experiments with radiolabelled TRP showed that labeled IAN enrichment is lower than that of labeled IAA, implying that IAN is not a direct product of IAoX [107, 108], and therefore there are likely to be other yet-to-be-defined reactions by which IAA is synthesized. In this regard, our microarray data reveal altered regulation of numerous genes encoding proteins of unknown function, including at least six cytochrome P450s. Functional analysis of these genes may further refine our knowledge of auxin metabolism.

Lastly, FIL physically interacts with the LEUNIG/SEUSS co-repressor complex [40, 109], which also has been shown to interact with other regulators of floral development and inflorescence architecture (e.g. AP1 and SEP3; [110]). Mutations in *SEUSS* (seu) and the *SEUSS-LIKE* (slk) genes condition auxin resistant growth phenotypes and exhibit reduced sensitivity to auxin [63, 111, 112]. Conversely, in gynoecia, *lug* and *seu* mutants exhibit increased sensitivity to inhibitors of polar auxin transport [113], and in *Antirrhinum*, mutations in the *LUG* homolog *STYLOSA* are associated with altered vascular development in leaves, hypersensitivity towards auxin and polar auxin transport inhibitors, and reduced polar auxin transport [109]. The *lug-1* mutant conditions a suppressed *bp er* phenotype that is similar to that of the *fil-10* suppressor (Figs 1B and 6A), suggesting that the two proteins may act cooperatively to coordinate inflorescence architecture through their influences on auxin biosynthesis, transport and perception.

### Glucosinolate metabolites can influence development and physiological processes

Our previous studies led us to postulate that BP acts to countermand the action of a vascular-borne growth repressor, but the nature of this signalling molecule has been elusive. Our observations that both *bp* and *fil* mutants alter glucosinolate profiles led us to consider the possibility that this repressor is linked to GSL metabolism. Evidence for this hypothesis is circumstantial but multifaceted. First, many genes involved in glucosinolate metabolism are predominantly expressed in vascular tissues and glucosinolates are known to be transported via the vasculature [114–116]. Second, indole-3-carbinol (I3C), a GSL breakdown product, has been shown to be an auxin antagonist, inhibiting auxin signalling and inducing growth arrest by interacting with the TIR1 auxin receptor [117, 118]. Third, although some molecules such as I3C are induced by herbivory, other GSL by-products are produced in unchallenged plants [119], and some are known to have growth inhibitory effects. Raphanusanin, generated from some GSL molecules by myrosinase action, is known to underpin blue light induced phototropism by inhibiting growth on the illuminated side of radish seedlings [120, 121], and exogenous application of raphanusanin in pea seedlings inhibits hypocotyl elongation and releases lateral buds from apical dominance [120, 122]. Our array analyses show that some hypothetical myrosinases are differentially expressed and could contribute to the generation of such inhibitory molecules. These genes represent intriguing targets for future functional genomics studies. Fourth, it is clear that glucosinolate metabolite levels can influence gene expression [123], as well as physiological processes such as flowering time [124–126]. Lastly, in seedlings treated with individually purified GLS molecules, changes in the transcriptome and developmental aberrations were observed (Kliebenstein lab, unpublished results). Collectively, these observations point to glucosinolate metabolites as contributors involved in fine tuning growth and development in addition to their well-established roles in orchestrating responses to biotic and abiotic stimuli.

## Supporting information

S1 Fig QRT-PCR analysis of GSL and auxin related genes in *bp er fil-10*. RNA from inflorescences of *bp er* and *bp er fil-10* was isolated and subjected to QRT-PCR. The fold change in *bp er fil-10* is shown. This is an independent experiment relative to the data presented in Figs 6 and 8.
(TIF)

S2 Fig Characterization of *bp er fil-4*. (A.) Inflorescence stem exhibiting a reduced floral cluster, consisting of type B flowerless pedicels (arrows). (B.) *bp er fil-4* inflorescence revealing the conversion of floral organs to filamentous structures. (C.) PCR analysis of RNA splicing. gDNA represents genomic Ler DNA, (-) is no DNA template reaction, and *bp er*, *bp er fil-4*, and *bp er fil-10* are cDNAs amplified from the relevant genotypes. DNA sequencing revealed that the *fil-4* mutation is due to a G to A base change at the exon 6 splice donor sequence. Note the congruence of the *bper* and *bperfil10* bands (337bp amplicon indicative of proper splicing of exon 5), and the larger 756bp amplicon in *bp er fil-4*, due to missplicing and the inclusion of intron 5 in the final mRNA. (D.) QRT-PCR analysis of glucosinolate metabolism genes. The expression pattern of these genes in the *fil-4* suppressor is different from that of the *fil-10* suppressor (see Figs 6 and 8), and the magnitude of the differences vs. the *bp er* parent line is much reduced. Elevated expression of myrosinases and CYP71A13 (CYP71) may provide avenues to shunt glucosinolate intermediates to IAA biosynthesis. (E-G.) Glucosinolate profiling of L*er*, *bp er*, *bp er fil-4* and *bp er fil-10*. Graphs showing comparisons where Student’s T-tests reveal statistical significance are shown. (H.) T-test values for all pair-wise comparisons. Those with p-values of less than 0.05 are highlighted in grey. Note that I3M, which can be converted to IAA via myrosinase/nitrilase activities, is elevated (see Fig 8 for pathway).
(TIF)

S3 Fig Expression of *STM*, *KNAT2* and *KNAT6* is unchanged in *bp er fil-10*. QRT-PCR of *bp er* and *bp er fil-10* inflorescence RNA reveals no significant changes in the expression of these *KNOX* genes in the two genotypes.
(TIF)

S1 Table List of primer sequences and information. (PDF)

S2 Table Genes up-regulated in *bp er fil-10*. (XLSX)

S3 Table Genes down-regulated in *bp er fil-10*. (XLSX)
