# Supplementary figures and images for: A novel Filamentous Flower mutant suppresses brevipedicellus developmental defects and modulates glucosinolate and auxin levels
Source: PLoS One. 2017 May 11;12(5):e0177045. doi: 10.1371/journal.pone.0177045 (PMC5426679; doi:10.1371/journal.pone.0177045)

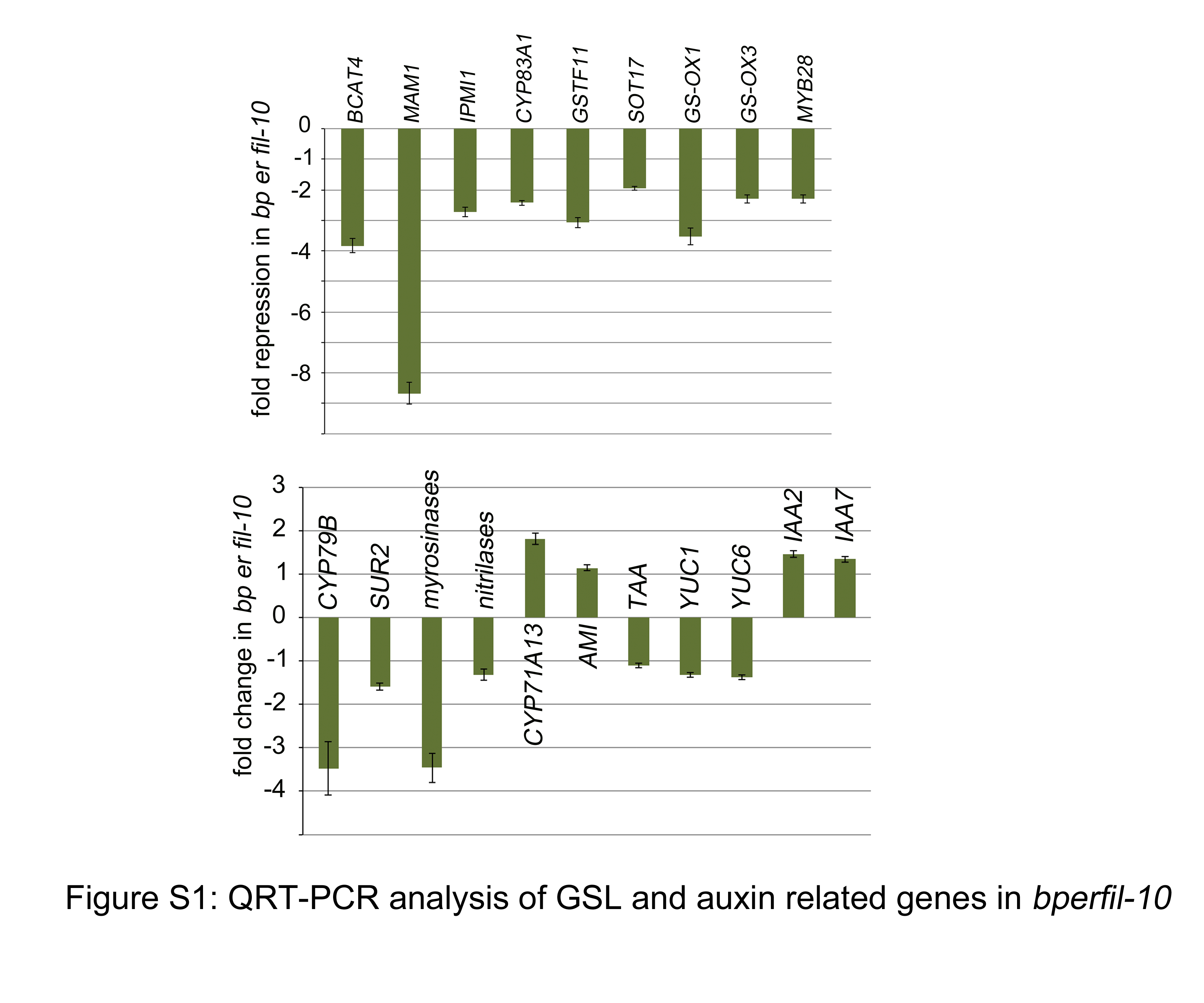

Supplement: S1 Fig — RNA from inflorescences of bp er and bp er fil-10 was isolated and subjected to QRT-PCR. The fold change in bp er fil-10 is shown. This is an independent experiment relative to the data presented in Figs 6 and 8. (TIF) [file pone.0177045.s001.tif]

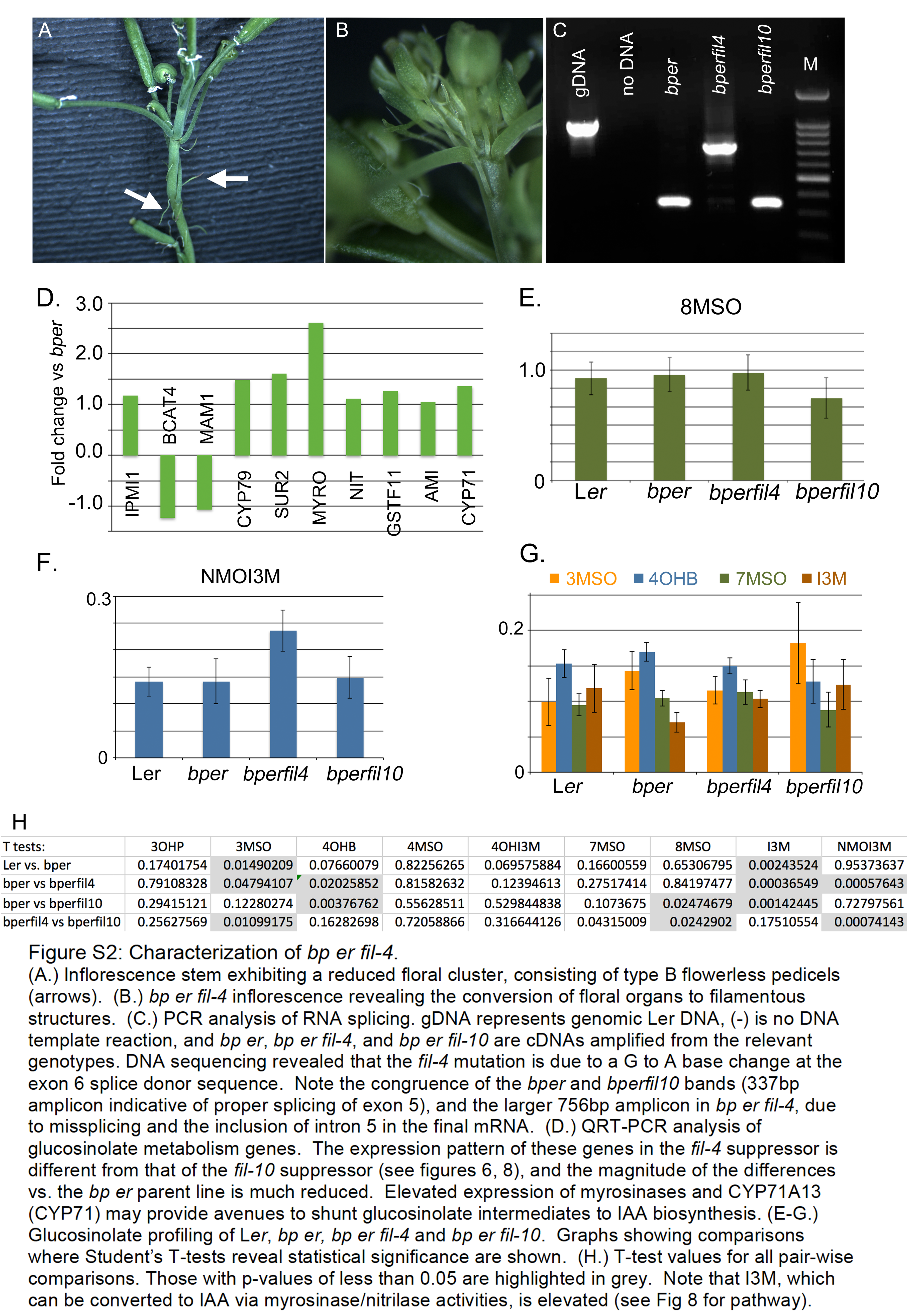

Supplement: S2 Fig — (A.) Inflorescence stem exhibiting a reduced floral cluster, consisting of type B flowerless pedicels (arrows). (B.) bp er fil-4 inflorescence revealing the conversion of floral organs to filamentous structures. (C.) PCR analysis of RNA splicing. gDNA represents genomic Ler DNA, (-) is no DNA template reaction, and bp er, bp er fil-4, and bp er fil-10 are cDNAs amplified from the relevant genotypes. DNA sequencing revealed that the fil-4 mutation is due to a G to A base change at the exon 6 splice donor sequence. Note the congruence of the bper and bperfil10 bands (337bp amplicon indicative of proper splicing of exon 5), and the larger 756bp amplicon in bp er fil-4, due to missplicing and the inclusion of intron 5 in the final mRNA. (D.) QRT-PCR analysis of glucosinolate metabolism genes. The expression pattern of these genes in the fil-4 suppressor is different from that of the fil-10 suppressor (see Figs 6 and 8), and the magnitude of the differences vs. the bp er parent line is much reduced. Elevated expression of myrosinases and CYP71A13 (CYP71) may provide avenues to shunt glucosinolate intermediates to IAA biosynthesis. (E-G.) Glucosinolate profiling of Ler, bp er, bp er fil-4 and bp er fil-10. Graphs showing comparisons where Student’s T-tests reveal statistical significance are shown. (H.) T-test values for all pair-wise comparisons. Those with p-values of less than 0.05 are highlighted in grey. Note that I3M, which can be converted to IAA via myrosinase/nitrilase activities, is elevated (see Fig 8 for pathway). (TIF) [file pone.0177045.s002.tif]

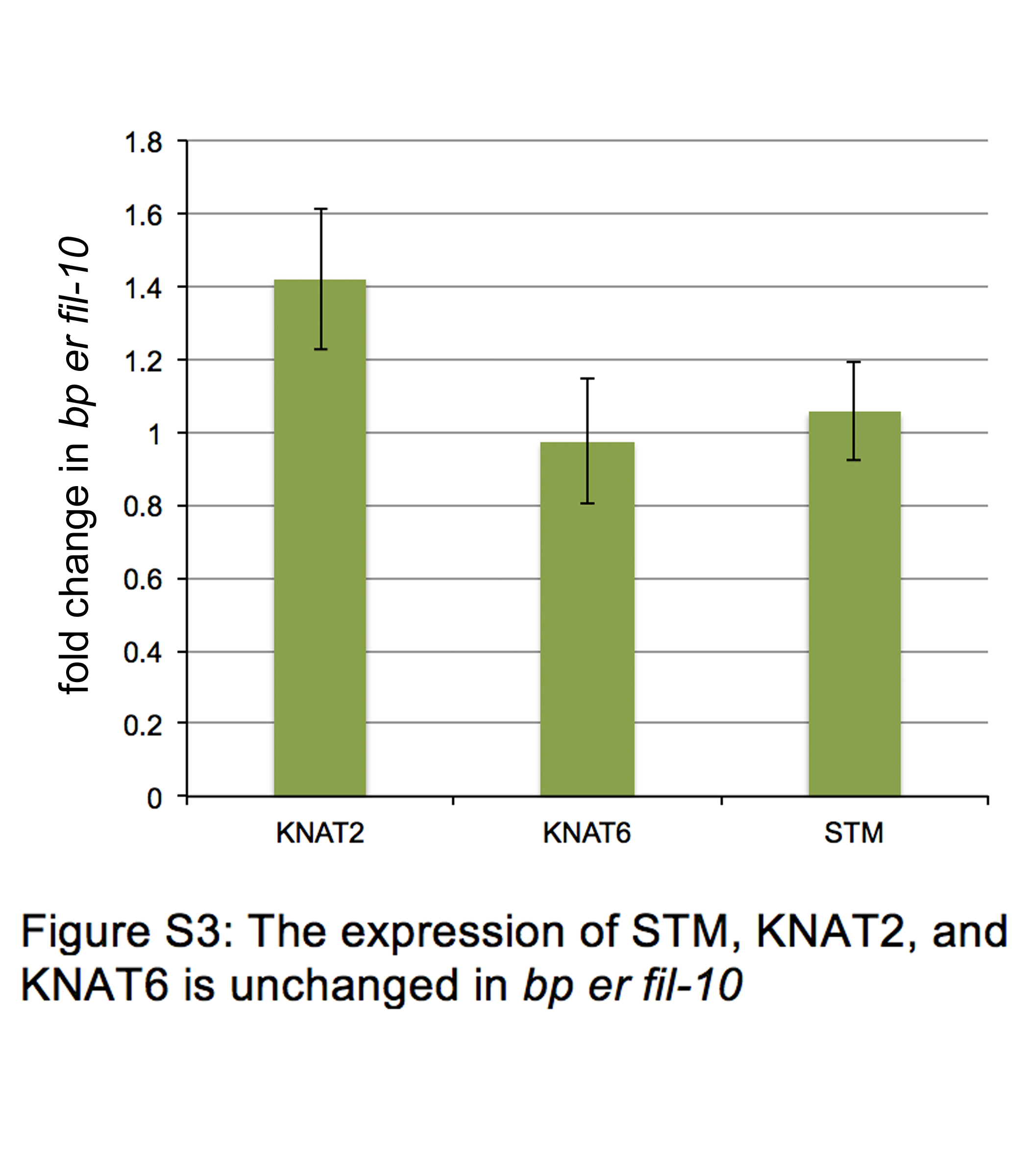

Supplement: S3 Fig — QRT-PCR of bp er and bp er fil-10 inflorescence RNA reveals no significant changes in the expression of these KNOX genes in the two genotypes. (TIF) [file pone.0177045.s003.tif]
