# Supplementary material for: A novel Filamentous Flower mutant suppresses brevipedicellus developmental defects and modulates glucosinolate and auxin levels
Source: PLoS One. 2017 May 11;12(5):e0177045. doi: 10.1371/journal.pone.0177045 (PMC5426679; doi:10.1371/journal.pone.0177045)
Supplement: S1 Table — (PDF) [file pone.0177045.s004.pdf]

Supplementary Table 1: Primer sequences

List of primers used to sequence the *FIL* gene

Primer name	Strand polarity	Sequence	Location vs. start codon
FILproFOR	Plus	TTCAAATGGTGTTTAGGGTGTAGTTCCGAAAC	-664
FIL FOR	Plus	AAAAGATGTCTATGTCGTCTATGTCCTCC	-4
FIL FOR2	Plus	TTCGCTTTGTCTTTGCCGTTGAATTTC	1162
FIL BACK2	Minus	AGACGGTGCATCTCTCAGCTCCTC	515
FIL BACK	Minus	GAATCGGTTATATGCGGATGGGACTC	1243
FIL 3' UTR	Minus	GGTTTACCTGTTGGGGCATGTTGG	1917

List of primers used to genotype/sequence mutant alleles

Gene	Primer name	Sequence	Mutant notes
FIL	FILgenotypeFOR	GTCAAATACCCATCAATCAATCAAATCC	*fil-10*: C to T change creates HpaII site for CAPS analysis
	FILgenotypeBACK	AGACGGTGCATCTCTCAGCTCCTC	
	FILgenotypeFOR3	AGTCCCATCCGCATATAACCGATT	*fil-4*: T to A mutation confirmed by sequencing with FOR3 primer
	FILgenotypeBACK2	ACCACAACTTTTGGACATGATAAACCC	
YAB3	YAB3genotypeFOR<br>YAB3 BACK4	ACCACATCTTCCTCTCCACCGTTC<br>TCTGACCGTCACCGTCTTGA	509bp amplicon for wildtype
	Ds5-1	CCGTTTACCGTTTTGTATATCCCG	*yab3-2*:Paired with YAB3 BACK4 to give ~200bp amplicon for transposon insertion line yab3-2.
LAS	LASgenotype FOR	ACGGTGTTCACGAGCAGTGTATG	*las-11*: G to A mutation confirmed by sequencing with FOR primer
	LASgenotype BACK	AGAAGAGGGGACGATTTTGCCAG	
AS2	AS2genotypeFOR	CAATGGCGGCTTTGTGTAGG	*as2-101*: G to T change creates HpaII site for CAPS analysis
	AS2genotypeBACK	GATAGTGGTCTCTCACGGCGGT	
KAN1	KAN1genotype FOR	CACAACCGTTCATTCCCTTTCC	*kan1-1*: C to T change abolishes Sau3A site for CAPS analysis
	KAN1genotype BACK	CCCATTTCTTCTTCTCCTGACCC	

KAN2	KAN2genotype FOR	GGTTCATCATCTGTGGAAACCGACTG	
	KAN2genotype BACK	CTCAAGAACTGATTTAGGTGTTGCTCC	
	KAN2sequencing	CCACCAAACAGTAAACCATCATCG	*kan2-1:* Used to sequence mutant

List of Primers used for QRT-PCR

Primer name	Sequence	Amplicon size
PP2Ag FOR	TTCTCGAAACGCTTGATGTG	117
PP2Ag BACK	TTGCATCAGTCATAGGCGAG	
ACT7 FOR	GGAGCTGAGAGATTCCGTTG	285
ACT7 BACK	GATTGATCCTCCGATCCAGA	
UGT74B FOR	GTGAAAGGCCACGTCGTAAT	183
UGT74B BACK	GAAATCGAATCCATCGGAGA	
IPMI2 FOR	GAATCCGACTCTAACGAAGC	374
IPMI2 BACK	GGAAACACCTCTCCTGTAGC	
AMI FOR	AGCTGCTCGTCTTGTGGATT	164
AMI BACK	CAAACCATCCAACTGTGTCG	
GS-OX1 FOR	GAGTGGTATGCAACGCTTGA	171
GS-OX1 BACK	GTGGGAGACCATTCTCTGGA	
GS-OX3 FOR	GAATGGGACGACGATGAACT	165
GS-OX3 BACK	TTGAAGGCTTGGAATCAACC	
SOT17 FOR	ACGATGAGAGCTGATCCTTT	166
SOT17 BACK	CTCTGTCTTTCTCCCCTTTG	
CYP83A FOR	GAGGAAGAGGCTAGGAGGAT	334
CYP83A BACK	TAGGATCAAGCGTCTCATTG	
MAM1 FOR	GTTGCTCTTCTGTGTCCAAA	313
MAM1 BACK	GTTTCCTCATCCACCTCATT	
CYP71A13 FOR	ATTCGGATCAGGGAGAAGGATA	152
CYP71A13 BACK	CGATACCAATGGCTTCAGTTAGAT	
CYP79B2 FOR	TACCGGGAAAAGAGGTTGTG	197
CYP79B2 BACK	AGAGATGCTCCGGCAATCTA	
BCAT4 FOR	CAAGTTGCTCTTGCCAACAA	212
BCAT4 BACK	GGAAAAGCTCGACGAAACTG	
CYP81F4 FOR	TCATCAGAGACCTCCGCTTT	261
CYP81F4 BACK	GTCACGTGGCACATCGTATC	
IAA2 FOR	ATCAACCAGCTCACCAAGAACAA	179
IAA2 BACK	CGGGTAATCCAAGACATAGCTC	
IAA7 FOR	CTGCTGTTCCCAAGGAGAAG	284
IAA7 BACK	GGCCAATGCATCAGAAAGAT	
IAA19 FOR	TGGTGGTGACGCTGAGAAGG	146
IAA19 BACK	TGCTCACTTTCACATACCCTAACC	

GSTF11 FOR	AGTATGCGGACCAAGGAACG	114
GSTF11 BACK	CTAAGGGTAGAGCCACAGCG	
MYB28 FOR	ACTGCGATGGACCAACTACC	251
MYB28 BACK	CGACCGTAGGGTTGGAACTA	
STM FOR	TTAGGGAGCCTCAAGCAAGA	109
STM BACK	AAGGCCATTTGTAGTGACGG	
KNAT2 FOR	GCTCGACGGAAGACTATTCG	127
KNAT2 BACK	GACAAAGCCGGTAGCGAGTTC	
SUR2 FOR	CGTCATGACTGGACTCGCTA	116
SUR2 BACK	TGGTCACGCCATATCTACCA	
TAA1 FOR	GATGAAGAATCGGTGGGAGA	236
TAA1 BACK	TGACTCGGACATGCTTCTTG	
LAX1 FOR	ATTCGGATCAGTCATCCAGC	280
LAX1 BACK	GGTGGCTCCGGTAAAGTACA	
MYROSINASES FOR	GGACCAGTGATGATAACTAGATGGTTTCT	247[1]
MYROSINASES BACK	TGGGCGTATTGAGTGACGTAATAGTTG	
TGG1 FOR	GCAGCCAGTTACTACTACCCAAA	248
TGG1 BACK	TTATCCCCAAGAGACCAAGC	
NITRILASES FOR	GTTCATAACGAAGAAGGGCGTG	279[2]
NITRILASES BACK	TGTCGTAAACGGGGATGGTTG	
YUC1 FOR	GAACACCGTTCATGTGTTGC	206
YUC1 BACK	GACTTTTGCCGGTGACATTT	
YUC6 FOR	GAAGGCACGACGGAGTATGT	228
YUC6 BACK	AGCACCGAAGTTGCAGAGAT	

[1]Myrosinase cDNAs (TGG1 and TGG2) were both amplified by this primer set; their sequence lengths and thus melting temperatures were identical.
[2]Nitrilase cDNAs (NIT1, NIT2, and NIT3) were amplified by this primer set; product lengths were identical and the products' melt temperature differed by less than 1°C.

Primers used for multiplex PCR to determine relative DR5 gene copy number in transgenic lines. Annealing was conducted at 58°C.

Primer name	Sequence	Amplicon size
EGADjunctionFOR	CATGAGCGGAGAATTAAGGGAGTC	712bp
GUSgenotypeBACK	GACCCACACTTTGCCGTAATGAGTG	
AMIgenotypeFOR	GAGTGCACATTGTCTTTAGGATGGTTTGCTCGG	306bp
AMIgenotypeBACK	TCACCGGTTTTACTACAGTGTTGCCTTGATACAGAAC	

Primers used to amplify the region of FIL containing the *fil-4* lesion and to examine the splicing defect

Primer name	Sequence	Amplicon size
FILgenotype FOR3	AGTCCCATCCGCATATAACCGATT	337bp WT<br>756bp *fil-4*
FILgenotypeBACK2	ACCACAACTTTTGGACATGATAAACCC	
